# Synthesis of 1-(5-Chloro-2-hydroxyphenyl)-5-oxopyrrolidine-3-carboxylic Acid Derivatives and Their Antioxidant Activity

**DOI:** 10.3390/molecules24050971

**Published:** 2019-03-09

**Authors:** Ingrida Tumosienė, Kristina Kantminienė, Ilona Jonuškienė, Artūras Peleckis, Sergey Belyakov, Vytautas Mickevičius

**Affiliations:** 1 Department of Organic Chemistry, Kaunas University of Technology, LT-50254 Kaunas, Lithuania; ingrida.tumosiene@ktu.lt (I.T.); ilona.jonuskiene@ktu.lt (I.J.); arturaspeleckis@gmail.com (A.P.); vytautas.mickevicius@ktu.lt (V.M.); 2 Department of Physical and Inorganic Chemistry, Kaunas University of Technology, LT-50254 Kaunas, Lithuania; 3 Laboratory of Physical Organic Chemistry, Latvian Institute of Organic Synthesis, Aizkraukles 21, 1006 Riga, Latvia; serg@osi.lv

**Keywords:** pyrrolidin-2-one, azoles, antioxidative, X-ray

## Abstract

A series of novel 1-(5-chloro-2-hydroxyphenyl)-5-oxopyrrolidine-3-carboxylic acid derivatives containing chloro, hydroxyl, isopropyl, nitro, nitroso, and amino substituents at benzene ring and 1-(5-chloro-2-hydroxyphenyl)-5-oxopyrrolidine-3-carbohydrazide derivatives bearing heterocyclic moieties were synthesized. Antioxidant activity of the synthesized compounds was screened by DPPH radical scavenging method and reducing power assay. A number of compounds were identified as potent antioxidants. Antioxidant activity of 1-(5-chloro-2-hydroxyphenyl)-4-(5-thioxo-4,5-dihydro-1,3,4-oxadiazol-2-yl)pyrrolidin-2-one has been tested to be 1.5 times higher than that of a well-known antioxidant ascorbic acid. 1-(5-Chloro-2-hydroxyphenyl)-4-(4-methyl-5-thioxo-4,5-dihydro-1*H*-1,2,4-triazol-3-yl)pyrrolidin-2-one has shown 1.35 times higher antioxidant activity than that of vitamin C by DPPH radical scavenging method and optical density value of 1.149 in reducing power assay. The structure of 1-(5-chloro-2-hydroxyphenyl)-*N*-(1,3-dioxoisoindolin-2-yl)-5-oxopyrrolidine-3-carboxamide was unambiguously assigned by means of X-ray diffraction analysis data.

## 1. Introduction

Antioxidants are compounds slowing down or inhibiting the oxidation processes which occur under the influence of reactive oxygen species (ROS) produced by living organisms as a result of normal cellular metabolism and environmental factors. ROS, including superoxide anion (O_2_^−^), peroxyl (ROO^−^) radicals and reactive hydroxyl (OH^−^) as well as hydrogen peroxide (H_2_O_2_), are highly reactive and can damage cell structures such as carbohydrates, nucleic acids, lipids, and proteins and alter their functions [1]. They are used for stabilization of food products, polymeric products, petrochemicals, cosmetics, and pharmaceuticals. Antioxidants are involved in the defense mechanism of the organism against pathologies associated with the attack of free radicals [2]. Oxidative stress deregulates a series of cellular functions and is involved in various pathological conditions such as AIDS, arthritis, asthma, autoimmune diseases, carcinogenesis, cardiovascular dysfunction, cataract, diabetes, neurodegenerative diseases, Alzheimer’s disease, Parkinson’s dementia, etc [3].

Recently, scientists in various disciplines have been extensively investigating new compounds, either natural or synthesized ones, as potential antioxidants capable of preventing or reducing the impact of oxidative stress on cells. 2-Pyrrolidinone rich fraction of *Brassica oleracea var. capitata* has been shown to exhibit antioxidant and in vitro anticancer activities [4]. 8-C *N*-Ethyl-2-pyrrolidinone substituted flavan-3-ols isolated from the aqueous extract of pu-erh tea have been proven to possess antioxidant activity [5]. Pyrrole derivatives comprise a class of biologically active heterocyclic compounds which can serve as promising scaffolds for agents possessing antioxidant [3,6,7], anticancer, anti-depressant, anti-inflammatory, anti-malarial, anti-HIV, antibacterial [8], anti-tubercular, anti-ulcer, anti-hypertensive, insecticidal, cytotoxic, and antiviral [9,10] properties.

Besides well-known antioxidants, phenolic compounds, a number of other class compounds have been identified as possessing antioxidant activity. Thiol- [11] and amine-containing [12] compounds are among them, making different heterocycles attractive scaffolds for the development of new antioxidant agents. Derivatives bearing 1,3,4-thiadiazole [13] and pyrazole [14,15] moieties have been recently demonstrated to possess antioxidant activity. Owing to their extremely wide spectrum of biological properties, 1,2,4-triazole derivatives are of a medical and pharmaceutical interest. Compounds with 1,2,4-triazole nucleus have been evaluated as possessing antimicrobial, antitumor, antitubercular, anticonvulsant, antidepressant, antimalarial, anti-inflammatory, etc. activities [14,16,17,18,19,20].

As a continuation of our search for the nitrogen-containing heterocyclic compounds exhibiting antioxidative properties [21,22], we report herein the synthesis of a series of 1-(5-chloro-2-hydroxyphenyl)-5-oxopyrrolidine-3-carboxylic acid derivatives bearing heterocyclic moieties.

## 2. Results and Discussion

### 2.1. Chemistry

**Scheme 1:**
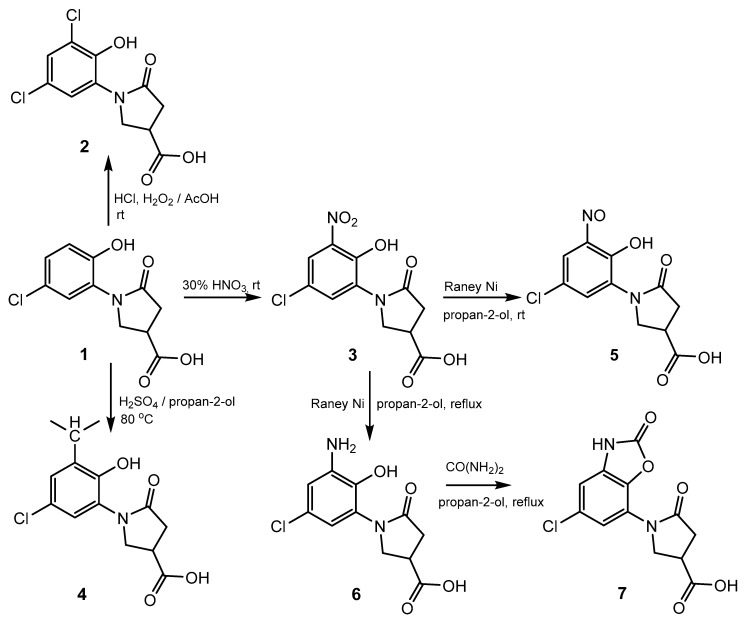
Synthesis of compounds **2**–**6**.

Reaction of 1-(5-chloro-2-hydroxyphenyl)-5-oxopyrrolidine-3-carboxylic acid (**1**) [23] with 6 M hydrochloric acid in the presence of acetic acid at room temperature followed by treatment with hydrogen peroxide for 12 h provided acid **2** bearing two chlorine substituents in the benzene ring (Scheme 1).

Nitration reaction of **1** in 30% nitric acid at room temperature gave derivative **3**. An isopropyl substituent was introduced into position 3 of the benzene ring of **1** by treating it with propan-2-ol in sulphuric acid to give acid **4**. Reaction of **3** in the presence of Raney Ni in propan-2-ol at room temperature led to reduction of nitro group to nitroso one in **5**, whereas reduction at reflux temperature provided amino derivative **6**. Structures of the synthesized compounds have been confirmed by the ^1^H and ^13^C-NMR spectra and HRMS data (Supplementary Materials, Figures S1–S77). Presence of the amino group is indicated by a singlet at 4.95 ppm in the ^1^H-NMR spectrum of **6**, whereas two OH groups have resonated as a broad singlet at 8.00 ppm. The resonances of aromatic protons are shifted upfield to 6.29 ppm and 6.55 ppm owing to the influence of the NH_2_ group. Reaction of **6** with urea in propan-2-ol provided 1-(5-chloro-2-oxo-2,3-dihydrobenzo[*d*]oxazol-7-yl)-5-oxopyrrolidine-3-carboxylic acid (**7**). In the ^1^H-NMR spectrum for **7**, the broad singlet at 12.00 ppm attributed to the NH group proton has proven the formation of benzoxazole moiety. New heterocyclic ring has influenced carbon resonances of the pyrrolidinone ring and the signal of the CH_2_N group shifted upfield to 50.5 ppm in comparison with the ^13^C-NMR spectrum for **6**.

**Scheme 2:**
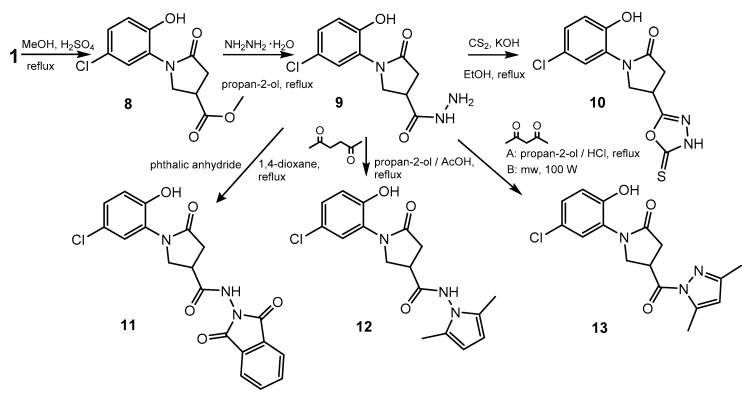
Synthesis of compounds **8**–**13**.

Methyl 1-(5-chloro-2-hydroxyphenyl)-5-oxopyrrolidine-3-carboxylate (**8**) was prepared from acid **1** by the usual esterification procedure of carboxylic acids (Scheme 2). Ester **8** was converted to hydrazide **9** by the reaction with hydrazine hydrate in propan-2-ol at reflux temperature of the reaction mixture.

Hydrazide **9** was used as a precursor for the synthesis of a number of heterocyclic derivatives. By employing the convenient reaction of acid hydrazides with carbon disulfide in alcohol in the presence of KOH [24,25], 1-(5-chloro-2-hydroxyphenyl)-4-(5-thioxo-4,5-dihydro-1,3,4-oxadiazol-2-yl)pyrrolidin-2-one (**10**) was synthesized from hydrazide **9** with carbon disulfide followed by cyclization in situ of the formed potassium salt of hydrazinocarbothioate with hydrochloric acid to pH 3–4. Formation of oxadiazole ring in **10** has been confirmed by the presence of carbon resonances at 164.1 ppm (C=N) and 174.2 ppm (C=S) in the ^13^C-NMR spectrum. Reaction of **9** with phthalic anhydride in 1,4-dioxane at reflux temperature of the reaction mixture provided phthalimide derivative **11**. In its ^1^H-NMR spectrum, multiplet in the range of 7.93–8.00 ppm has been ascribed to the benzene ring protons in the phthalimide moiety. The proton of the NH group is deshielded by 0.7 ppm in comparison with the one in **9**.

Condensation reactions of acid hydrazides with aliphatic diketones provide five-membered heterocyclic compounds [25,26]. Thus, pyrrole derivative **12** was obtained from hydrazide **9** by refluxing it with hexane-2,4-dione in propan-2-ol in the presence of acetic acid for 2 h. Reaction of **9** with pentane-2,5-dione in the presence of concentrated HCl at reflux temperature afforded pyrazole derivative **13** in 82% yield over 5 h. The reaction was much faster (20 min), when the reaction mixture was subjected to microwave irradiation at 100 °C. However, the yield of the target product was lower and reached 60%.

**Scheme 3:**
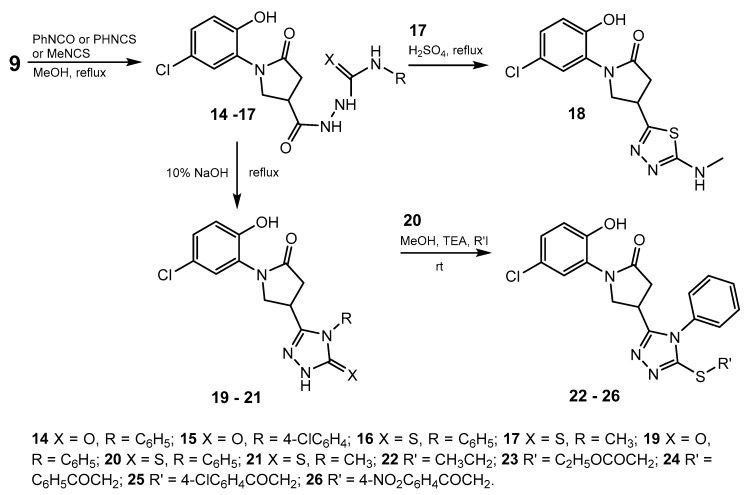
Synthesis of compounds **14**–**26**.

Reaction of hydrazide **9** with various isocyanates or isothiocyanates in methanol led to the formation of carboxamides **14**, **15**, and carbothioamides **16**, **17**, respectively (Scheme 3).

Absorption bands of carbonyl groups in the region of 1694–1652 cm^−1^ and absorption band of the C=S group at 1206 cm^−1^ (**16**) and 1255 cm^−1^ (**17**) supported the structure of carbothioamides **16** and **17**. 1-(5-Chloro-2-hydroxyphenyl)-4-(5-(methylamino)-1,3,4-thiadiazol-2-yl)pyrrolidin-2-one (**18**) was synthesized by condensation reaction of carbothioamide **17** in acidic medium provided by sulphuric acid. Carbon resonances in the ^13^C-NMR spectrum for **18** at 152.9 ppm (CS) and 167.4 ppm (CNH) have confirmed formation of the thiadiazole moiety. Condensation reactions of carboxamide **14** and carbothioamides **16** and **17** in alkaline medium furnished triazolone **19** and triazolethiones **20** and **21**, respectively. In the ^13^C-NMR spectra of **20** and **21**, signals at approx. 171–172 ppm have been ascribed to the carbon of C=S group. Alkylation reactions of triazolethione **20** under basic conditions, when its thione–thiol equilibrium is shifted towards the formation of thiole [14], provided *S*-alkylated derivatives **22**–**26**. In the ^1^H-NMR spectra of the synthesized S-substituted derivatives 22–26, protons of the SCH_2_ group resonated in the range of 3.1–4.91 ppm. In the ^13^C-NMR spectra for **24**–**26**, carbon resonances at approx. 192 ppm have been ascribed to the carbon in carbonyl group adjacent to benzene ring.

### 2.2. Crystal Structure of Compound ***11***

**Figure 1:**
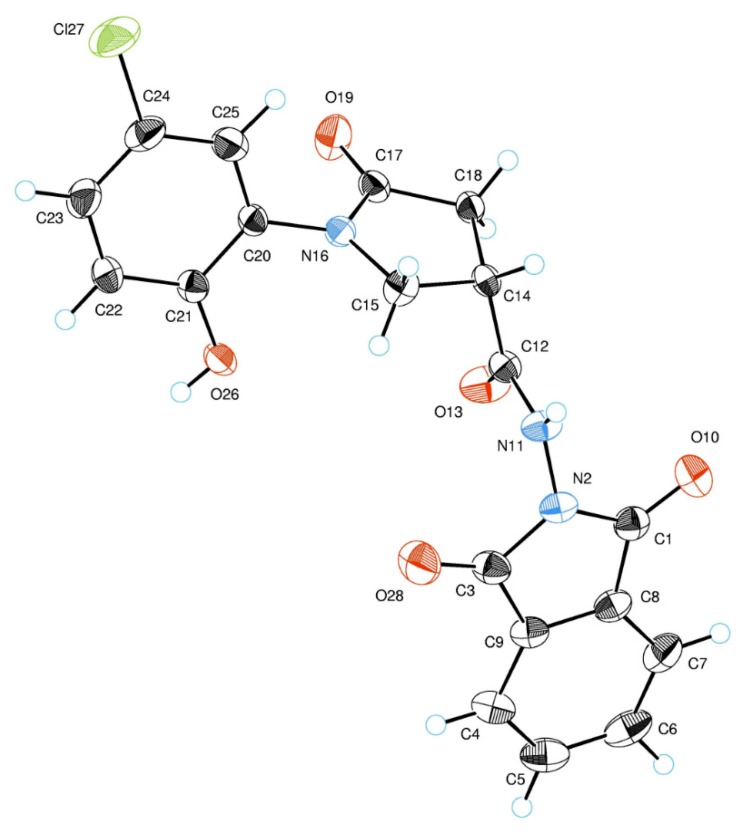
ORTEP (Oak Ridge Thermal-Ellipsoid Plot Program) molecular structure of **11**, showing the atom-numbering scheme. Displacement ellipsoids are drawn at the 50% probability level and hydrogen atoms are shown as small spheres of arbitrary radii.

The structure of **11** was examined in more detail. Figure 1 shows a perspective view of molecule **11** with thermal ellipsoids and the atom-numbering scheme followed in the text. The pyrrolidin-2-one cycle is planar and forms with the phenyl ring of C20−C21−C22−C23−C24−C25 dihedral angle of 71.2(3)°. In the molecular structure, the fragment of HN11−C12−O13 is almost perpendicular to both pyrrolidin-2-one cycle (dihedral angle is 80.2(3)°) and isoindol-1,3-dione system (dihedral angle is equal 86.0(3)°).

**Figure 2:**
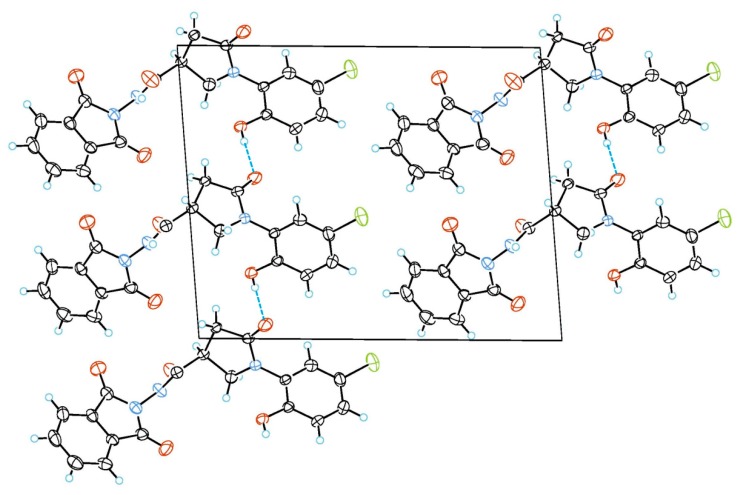
A projection of the crystal structure of **11** along monoclinic axis.

Figure 2 illustrates a projection of the crystal structure along monoclinic axis. The strong intermolecular hydrogen bonds of OH···O type are observed between hydroxyl group O26−H and carbonyl oxygen O19 with 2.615(3) Å (H···O19 = 1.79(5) Å, O26−H···O19 = 165(3)°) length. By means of these bonds, the molecular chains along crystallographic direction [100] are formed in the crystal structure. There are also intermolecular hydrogen bonds of NH···O type between N11−H and carbonyl oxygen O13 forming in the crystals. The length of these bonds is 2.792(3) Å (H···O13 = 1.84(2) Å, N11−H···O19 = 162(3)°). The chains along monoclinic axis are formed in the crystal structure by means of these bonds.

### 2.3. Antioxidant Activity

Antioxidant activity of the synthesized compounds **1**–**26** was screened by the 2,2-diphenyl-1-(2,4,6-trinitrophenyl)hydrazyl (DPPH) radical scavenging method and reducing power assay. DPPH assay is considered to be an accurate, easy, and economic method to evaluate radical scavenging activity of antioxidants, since the radical compound is stable and need not be generated [27]. In this method, the antioxidant efficiency is measured at ambient temperature, therefore the risk of thermal degradation of the tested molecules is eliminated.

As seen from the results presented in Table 1, compounds **10** (88.6%), **19** (87.7%), and **21** (78.6%) possess a very high DPPH radical scavenging ability. Introduction of 1,3,4-oxadiazole moiety at position 3 of 5-oxopyrrolidine ring led to 1.5 times higher antioxidant activity of **10** in comparison with that of a well-known antioxidant ascorbic acid used as a positive control. Similar activity was detected for compound **19**, bearing the phenyl substituent at position 4 of triazolone ring, whereas its thio analogue **20** demonstrated somewhat lower activity (58.4%), though it is close to the effect of ascorbic acid. Replacement of the phenyl substituent by the methyl one in the 1,2,4-triazole-3-thione derivative **21** led to 1.35 times higher antioxidant activity in comparison with that of vitamin C. Analysis of a structure–activity relationship for *S*-substituted 4-phenyl-1,2,4-triazoles **22**–**26** has revealed that *S*-alkyl substituents did not have notable influence on the antioxidant properties of the compounds, whereas introduction of phenyl fragment led to significantly higher DPPH radical scavenging ability. Antioxidant activity of compound **26** bearing a *p*-nitrophenyl fragment was the highest (66.8%) among the compounds of this group.

In the reducing power assay, the presence of reducing agents (antioxidants), which donate an electron, in a sample results in the reduction of Fe^3+^ to Fe^2+^. The amount of the Fe^2+^ complex can then be monitored by measuring the formation of Perl’s blue at 700 nm. The results of the reducing power assay (Table 1) show that the highest antioxidant effect has been demonstrated by 5-oxopyrrolidine derivatives containing a free carboxylic moiety. 1-(3-amino-5-chloro-2-hydroxyphenyl)-5-oxopyrrolidine-3-carboxylic acid (**6**) (1.675) and its cyclic benzoxazole derivative **7** (1.573) were identified as the compounds possessing the strongest reducing properties. 1-(5-Chloro-2-hydroxyphenyl)-4-(4-methyl-5-thioxo-4,5-dihydro-1*H*-1,2,4-triazol-3-yl)pyrrolidin-2-one (**21**) was the only compound to exhibit high antioxidant activity in both different tests with an optical density value of 1.149 in the reducing power assay.

## 3. Experimental

### 3.1. General Information

Melting points were determined on a MEL-TEMP (Electrothermal, A Bibby Scientific Company, Burlington, NJ, USA) melting point apparatus and are uncorrected. FT-IR spectra (ν, cm^−1^) were recorded on a Perkin–Elmer Spectrum BX FT–IR spectrometer (Perkin–Elmer Inc., Waltham, MA, USA) using KBr pellets. ^1^H and ^13^C-NMR spectra were recorded in DMSO-*d_6_* on a Varian Unity Inova (300 MHz, 75 MHz) (Palo Alto, CA, USA) and Bruker Avance III (400 MHz, 101 MHz) (Bruker BioSpin AG, Fällanden, Switzerland) spectrometers operating in the Fourier transform mode. Chemical shifts (δ) are reported in parts per million (ppm) calibrated from TMS (0 ppm) as an internal standard for ^1^H-NMR, and DMSO-d_6_ (39.43 ppm) for ^13^C-NMR. Mass spectra were obtained on Bruker maXis UHR-TOF mass spectrometer (Bruker Daltonics, Bremen, Germany) with ESI ionization. The reaction course and purity of the synthesized compounds was monitored by TLC using aluminium plates precoated with silica gel 60 F_254_ (MerckKGaA, Darmstadt, Germany). Reagents were purchased from Sigma-Aldrich (St. Louis, MO, USA).

The microwave reactions were conducted using a CEM Discover Synthesis Unit (CEM Corp., Matthews, NC, USA). The microwave machine consists of a continuous focused microwave power delivery system with operator-selectable power output from 0 to 300 W. The reaction was performed in glass vessels (capacity 10 mL) sealed with a septum. The pressure was controlled by a load cell connected to the vessel. The temperature of the contents of the vessel was monitored using a calibrated infrared temperature control mounted under the reaction vessel. All experiments were performed using a stirring option whereby the contents of the vessel are stirred by means of a rotating magnetic plate located below the floor of the microwave cavity and a Tefloncoated magnetic stir bar in the vessel.

### 3.2. Synthesis

*1-(5-Chloro-2-hydroxyphenyl)-5-oxopyrrolidine-3-carboxylic acid* (**1**) [23]: A mixture of itaconic acid (97.57 g, 0.75 mol) dissolved in water (200 mL) and 2-amino-4-chlorophenol (71.79 g, 0.5 mol) was heated at reflux for 24 h. Afterwards, it was cooled down to room temperature and diluted with aqueous 10% NaOH solution (300 mL). The solution was heated to the reflux temperature and cooled down to 20 °C. Then it was filtered and filtrate was acidified with HCl to pH 2. Precipitate was filtered off, washed with water, and recrystallized by dissolving in aqueous 5% NaOH solution (100 mL) and pouring into 10% HCl solution to pH 1. Yield 66%; beige solid; m.p. 176−177 °C; IR (KBr) ν_max_ (cm^−1^): 3174 (OH), 1740, 1637 (C=O); ^1^H-NMR and ^13^C-NMR were found to be identical with the ones described in [23]; HRMS (ESI): *m*/*z* calcd for C_11_H_11_ClNO_4_ 256.0376 [M + H]^+^, found 256.0374.

*1-(3,5-Dichloro-2-hydroxyphenyl)-5-oxopyrrolidine-3-carboxylic acid* (**2**): A mixture of 1-(5-chloro-2-hydroxyphenyl)-5-oxopyrrolidine-3-carboxylic acid (**1**) (0.26 g, 1 mmol), 6 M hydrochloric acid (10 mL), and acetic acid (4 mL) was stirred at room temperature for 2 h. Afterwards, 30% hydrogen peroxide (6 mL) was slowly added and stirring was continued for 12 h. Water (10 mL) was added, precipitate formed was filtered off, and recrystallized from ethanol. Yield 86%; beige solid; m.p. 189–190 °C; IR (KBr) ν_max_ (cm^−1^): 3375 (OH), 1710, 1667 (C=O); ^1^H-NMR (DMSO-*d_6_*, 400 MHz) δ: 2.59–2.71 (m, 2H, COCH_2_), 3.43 (qui, *J* = 8.0 Hz, 1H, CH), 3.84 (d, *J* = 8.4 Hz, 2H, NCH_2_), 7.27 (s, 1H, Ar-H), 7.48 (s, 1H, Ar-H), 10.03 (s, 1H, OH), 12.72 (br s, 1H, OH); ^13^C-NMR (DMSO-*d_6_*, 101 MHz) δ: 33.5 (CH), 36.3 (CH_2_), 50.8 (NCH_2_), 122.4, 122.5, 126.8, 128.1, 128.3, 148.5 (Ar-C), 172.8, 174.0 (C=O); HRMS (ESI): *m*/*z* calcd for C_11_H_10_Cl_2_NO_4_ 289.9987 [M + H]^+^, found 289.9985.

*1-(5-Chloro-2-hydroxy-3-nitrophenyl)-5-oxopyrrolidine-3-carboxylic acid* (**3**): A mixture of 1-(5-chloro-2-hydroxyphenyl)-5-oxopyrrolidine-3-carboxylic acid (**1**) (0.26 g, 1 mmol) and 30% HNO_3_ (10 mL) was stirred at room temperature until acid **1** completely dissolved. Precipitate formed after 10 min was filtered off, washed with water, and recrystallized from propan-2-ol. Yield 98%; yellow solid; m.p. 188–189 °C (propan-2-ol); IR (KBr) ν_max_ (cm^−1^): 3267 (OH), 1705, 1668 (C=O); ^1^H-NMR (DMSO-*d_6_*, 400 MHz) δ: 2.61–2.72 (m, 2H, COCH_2_), 3.44 (qui, *J* = 8.0 Hz, 1H, CH), 3.84–3.92 (m, 2H, NCH_2_), 7.68 (s, 1H, Ar-H), 7.97 (s, 1H, Ar-H), 10.96 (br s, 1H, OH), 12.76 (br s, 1H, OH); ^13^C-NMR (DMSO-*d_6_*, 101 MHz) δ: 33.4 (CH), 36.3 (CH), 50.7 (NCH_2_), 122.0, 123.4, 130.2, 133.5, 138.9, 147.0 (Ar-C), 172.9, 173.9 (C=O); HRMS (ESI): *m*/*z* calcd for C_11_H_10_ClN_2_O_6_ 301.0227 [M + H]^+^, found 301.0222.

*1-(5-Chloro-2-hydroxy-3-isopropylphenyl)-5-oxopyrrolidine-3-carboxylic acid* (**4**): A mixture of 1-(5-chloro-2-hydroxyphenyl)-5-oxopyrrolidine-3-carboxylic acid (**1**) (0.26 g, 1 mmol), 80% H_2_SO_4_, and propan-2-ol (10 mL) was stirred at 80 °C for 4 h. Afterwards, cold water (10 mL) was added. Precipitate was filtered off, washed with water, and recrystallized from propan-2-ol. Yield 90%; white solid; m.p. 158–159 °C; IR (KBr) ν_max_ (cm^−1^): 3140 (OH), 1718, 1664 (C=O); ^1^H-NMR (DMSO-*d_6_*, 400 MHz) δ: 1.15, 1.17 (2s, 6H, 2CH_3_), 2.66 (d, *J* = 8.4 Hz, 2H, COCH_2_), 3.25 (qui, *J* = 6.8 Hz, 1H, CH), 3.44 (qui, *J* = 8.4 Hz, 1H, CH), 3.79–3.89 (m, 2H, NCH_2_), 7.06 (s, 1H, Ar-H), 7.11 (s, 1H, Ar-H), 8.98 (br s, 2H, OH); ^13^C-NMR (DMSO-*d_6_*, 101 MHz) δ: 22.3 (CH_3_), 26.7 (CH), 33.8 (CH), 36.3 (CH_2_), 51.1 (NCH_2_), 122.6, 124.4, 124.9, 126.9, 138.8, 148.9 (Ar-C), 173.1, 174.2 (C=O); HRMS (ESI): *m*/*z* calcd for C_14_H_17_ClNO_4_ 298.0846 [M + H]^+^, found 298.0841.

*1-(5-Chloro-2-hydroxy-3-nitrosophenyl)-5-oxopyrrolidine-3-carboxylic acid* (**5**): To 1-(5-chloro-2-hydroxy-3-nitrophenyl)-5-oxopyrrolidine-3-carboxylic acid (**3**) (0.30 g, 1 mmol) dissolved in propan-2-ol (10 mL), Raney Ni (1 g) was added and a reaction mixture was stirred at room temperature for 3 h. Raney Ni was separated by filtration, the precipitate formed in a filtrate was filtered off, washed with water, and recrystallized from propan-2-ol. Yield 85%; khaki solid; m.p. 210–211 °C; IR (KBr) ν_max_ (cm^−1^): 3197 (OH), 1687(C=O); ^1^H-NMR (DMSO-*d_6_*, 400 MHz) δ: 2.66 (d, *J* = 8.8 Hz, 2H, COCH_2_), 3.45 (qui, *J* = 8.8 Hz, 1H, CH), 3.84–3.91 (m, 2H, NCH_2_), 7.66 (s, 1H, Ar-H), 7.95 (s, 1H, Ar-H), 11.74 (br s, 2H, OH); ^13^C-NMR (DMSO-*d_6_*, 101 MHz) δ: 33.3 (CH), 36.3 (CH_2_), 50.6 (NCH_2_), 121.7, 123.3, 130.2, 133.4, 138.9, 147.2 (Ar-C), 172.9, 173.9 (C=O); HRMS (ESI): *m*/*z* calcd for C_11_H_10_ClN_2_O_5_ 285.0278 [M + H]^+^, found 285.0472.

*1-(3-Amino-5-chloro-2-hydroxyphenyl)-5-oxopyrrolidine-3-carboxylic acid* (**6**): To 1-(5-chloro-2-hydroxy-3-nitrophenyl)-5-oxopyrrolidine-3-carboxylic acid (**3**) (1.20 g, 4 mmol) dissolved in propan-2-ol (15 mL), Raney Ni (8 g) was added and a reaction mixture was heated at reflux for 6 h. Raney Ni was separated by filtration, the filtrate was diluted with diethyl ether (15 mL), precipitate formed was filtered off and recrystallized from propan-2-ol. Yield 51%; beige solid; m.p. 159–160 °C; IR (KBr) ν_max_ (cm^−1^): 3268 (NH_2_), 3065 (OH), 1705 (C=O); ^1^H-NMR (DMSO-*d_6_*, 400 MHz) δ: 2.23–2.30 (m, 1H, COCH_2_), 2.68–2.74 (m, 1H, COCH_2_), 2.81–2.88 (m, 1H, CH), 3.61–3.68 (m, 2H, NCH_2_), 4.95 (s, 2H, NH_2_), 6.29 (s, 1H, Ar-H), 6.55 (s, 1H, Ar-H), 8.00 (br s, 2H, OH); ^13^C-NMR (DMSO-*d_6_*, 101 MHz) δ: 25.5 (CH), 36.3 (CH_2_), 52.3 (NCH_2_), 111.8, 114.1, 121.4, 125.0, 139.6, 139.8 (Ar-C), 173.6, 177.4 (C=O); HRMS (ESI): *m*/*z* calcd for C_11_H_12_ClN_2_O_4_ 271.0485 [M + H]^+^, found 271.0482.

*1-(5-Chloro-2-oxo-2,3-dihydrobenzo[d]oxazol-7-yl)-5-oxopyrrolidine-3-carboxylic acid* (**7**): To 1-(3-amino-5-chloro-2-hydroxyphenyl)-5-oxopyrrolidine-3-carboxylic acid (**6**) (0.16 g, 0.6 mmol) dissolved in propan-2-ol (20 mL), CO(NH_2_)_2_ (72 mg, 1.2 mmol) was added and a reaction mixture was heated at reflux for 4 h. Precipitate was filtered off, washed with water, and recrystallized from propan-2-ol. Yield 69%; brown solid; m.p. 153–154 °C; IR (KBr) ν_max_ (cm^−1^): 3131 (OH), 3050 (NH), 1765, 1730, 1693 (C=O); ^1^H-NMR (DMSO-*d_6_*, 400 MHz) δ: 2.64–2.80 (m, 2H, COCH_2_), 3.35–3.46 (m, 1H, CH), 4.03–4.15 (m, 2H, NCH_2_), 7.02 (s, 1H, Ar-H), 7.42 (s, 1H, Ar-H), 12.00 (br s, 1H, NH), 12.79 (b rs, 1H, OH); ^13^C-NMR (DMSO-*d_6_*, 101 MHz) δ: 33.7 (CH), 36.0 (CH_2_), 50.5 (NCH_2_), 107.0, 116.5, 122.3, 127.4, 132.6, 134.4 (Ar-C), 153.6, 172.0, 173.9 (C=O); HRMS (ESI): *m*/*z* calcd for C_12_H_10_ClN_2_O_5_ 297.0278 [M + H]^+^, found 297.0273.

*Methyl 1-(5-chloro-2-hydroxyphenyl)-5-oxopyrrolidine-3-carboxylate* (**8**): To 1-(5-chloro-2-hydroxyphenyl)-5-oxopyrrolidine-3-carboxylic acid (**1**) (12.75 g, 0.05 mol) completely dissolved in methanol (100 mL), H_2_SO_4_ (3 mL) was added, a reaction mixture was heated at reflux for 18 h. After cooling the reaction mixture down, aqueous 5% Na_2_CO_3_ solution was added until pH 7–8. Precipitate was filtered off, washed with water, and recrystallized from methanol. Yield 68%; white solid; m.p. 145−146 °C; IR (KBr) ν_max_ (cm^−1^): 3059 (OH), 1738, 1659 (C=O); ^1^H-NMR (DMSO-*d_6_*, 400 MHz) δ: 2.64–2.72 (m, 2H, COCH_2_), 3.48–3.52 (m, 1H, CH), 3.70 (s, 3H, CH_3_), 3.86–3.94 (m, 2H, NCH_2_), 6.93 (d, *J* = 9.1 Hz, 1H, Ar-H), 7.19 (d, *J* = 9.1 Hz, 1H, Ar-H), 7.24 (s, 1H, Ar-H), 9.97 (s, 1H, OH); ^13^C-NMR (DMSO-*d_6_*, 101 MHz) δ: 33.4 (CH), 36.0 (CH_2_), 50.4 (NCH_2_), 52.1 (OCH_3_), 118.1, 121.9, 126.5, 127.9, 151.8 (Ar-C), 172.0, 173.1 (C=O); HRMS (ESI): *m*/*z* calcd for C_12_H_13_ClNO_4_ 270.0533 [M + H]^+^, found 270.0542.

*1-(5-Chloro-2-hydroxyphenyl)-5-oxopyrrolidine-3-carbohydrazide* (**9**): To methyl 1-(5-chloro-2-hydroxyphenyl)-5-oxopyrrolidine-3-carboxylate (**8**) (8.07 g, 0.03 mol) completely dissolved in propan-2-ol (80 mL), hydrazine hydrate (1.92 g, 1.87 mL, 0.06 mol) was added. The reaction mixture was heated at reflux for 1.5 h. Precipitate was filtered off, washed with water, and recrystallized from methanol. Yield 66%; white solid; m.p. 193–194 °C; IR (KBr) ν_max_ (cm^−1^): 3360, 3302 (NH_2_), 3078 (OH), 2929 (NH), 1695, 1636 (C=O); ^1^H-NMR (DMSO-*d_6_*, 400 MHz) δ: 2.53–2.60 (m, 2H, COCH_2_), 3.18–3.23 (m, 1H, CH), 3.69–3.83 (m, 2H, NCH_2_), 4.32 (s, 2H, NH_2_), 6.92 (d, *J* = 9.1 Hz, 1H, Ar-H), 7.18 (d, *J* = 9.1 Hz, 1H, Ar-H), 7.22 (s, 1H, Ar-H), 9.28 (s, 1H, NH), 9.95 (s, 1H, OH); ^13^C-NMR (DMSO-*d_6_*, 101 MHz) δ: 34.2 (CH), 35.6 (CH_2_), 51.3 (NCH_2_), 118.0, 121.9, 126.6, 128.0, 151.8 (Ar-C), 171.6, 172.5 (C=O); HRMS (ESI): *m*/*z* calcd for C_11_H_13_ClN_3_O_3_ 270.0645 [M + H]^+^, found 270.0645.

*1-(5-Chloro-2-hydroxyphenyl)-4-(5-thioxo-4,5-dihydro-1,3,4-oxadiazol-2-yl)pyrrolidin-2-one* (**10**): To KOH (0.45 g, 8 mmol) dissolved in ethanol (25 mL), CS_2_ (0.91 g, 0.73 mL, 12 mmol) was added and a reaction mixture was stirred at room temperature for 15 min. Afterwards, 1-(5-chloro-2-hydroxyphenyl)-5-oxopyrrolidine-3-carbohydrazide (**9**) (1.08 g, 4 mmol) dissolved in ethanol (25 mL) was added and a reaction mixture was heated at reflux for 4 h. After solvent evaporation, the residue was dissolved in water (20 mL) and hydrochloric acid was added to pH 3–4. Liquid fractions were removed under vacuo and the residue was diluted with water. Precipitate was filtered off, washed with water, and recrystallized from methanol. Yield 79 %; white solid; m.p. 127–128 °C; IR (KBr) ν_max_ (cm^−1^): 3430 (OH), 2924 (NH), 1635 (C=O), 1118 (C=S); ^1^H-NMR (DMSO-*d_6_*, 400 MHz) δ: 2.59–2.66 (m, 2H, COCH_2_), 3.81–3.91 (m, 3H, CH+NCH_2_), 6.95–7.21 (m, 3H, Ar-H), 10.10 (s, 1H, OH), 13.56 (s, 1H, NH); ^13^C-NMR (DMSO-*d_6_*, 101 MHz) δ: 33.5 (CH), 36.2 (CH_2_), 50.6 (NCH_2_), 118.1, 121.8, 126.5, 127.9, 151.9 (Ar-C), 164.1 (C=N), 172.4 (C=O), 174.2 (C=S); HRMS (ESI): *m*/*z* calcd for C_12_H_11_ClN_3_O_3_S 312.0209 [M + H]^+^, found 312.0731.

*1-(5-Chloro-2-hydroxyphenyl)-N-(1,3-dioxoisoindolin-2-yl)-5-oxopyrrolidine-3-carboxamide* (**11**): To 1-(5-chloro-2-hydroxyphenyl)-5-oxopyrrolidine-3-carbohydrazide (**9**) (1.08 g, 4 mmol) dissolved in 1,4-dioxane (5 mL), phthalic anhydride (0.59 g, 4 mmol) was added and a reaction mixture was heated at reflux for 4 h. Precipitate was filtered off, washed with water, and recrystallized from propan-2-ol. Yield 87%; pink solid; m.p. 263–264 °C; IR (KBr) ν_max_ (cm^−1^): 3267 (OH), 3016 (NH), 1657, 1678, 1750 (C=O); ^1^H-NMR (DMSO-*d_6_*, 400 MHz) δ: 2.63–2.68 (m, 1H, COCH_2_), 2.75–2.79 (m, 1H, COCH_2_), 3.59 (qui, *J* = 4.6 Hz, 1H, CH), 3.80–3.84 (m, 1H, NCH_2_), 4.01–4.04 (m, 1H, NCH_2_), 6.93–7.24 (m, 3H, Ar-H), 7.93–8.00 (4H, m, Ar-H), 10.00 (s, 1H, NH), 10.96 (s, 1H, OH); ^13^C-NMR (DMSO-*d_6_*, 101 MHz) δ: 33.7 (CH); 35.0 (CH_2_); 50.9 (NCH_2_), 118.0, 121.9; 123.8, 126.4, 128.0, 129.5, 135.3, 151.8 (Ar-C), 165.0, 171.9, 171.9 (C=O); HRMS (ESI): *m*/*z* calcd for C_19_H_15_ClN_3_O_5_ 400.0700 [M + H]^+^, found 400.0693.

*1-(5-Chloro-2-hydroxyphenyl)-N-(2,5-dimethyl-1H-pyrrol-1-yl)-5-oxopyrrolidine-3-carboxamide* (**12**): A mixture of 1-(5-chloro-2-hydroxyphenyl)-5-oxopyrrolidine-3-carbohydrazide (**9**) (1.08 g, 4 mmol), hexane-2,5-dione (0.46 g, 0.47 mL, 4 mmol) propan-2-ol (40 mL), and glacial acetic acid (1 mL) was heated at reflux for 2 h. The reaction mixture was diluted with water (50 mL) and heated again up to boiling point. Precipitate formed after cooling down was filtered off, washed with water, and recrystallized from propan-2-ol. Yield 84%; white solid; m.p. 203–204 °C; IR (KBr) ν_max_ (cm^−1^): 3261 (NH), 3089 (OH), 1666, 1594 (C=O); ^1^H-NMR (DMSO-*d_6_*, 400 MHz) δ: 1.98, 2.00 (2s, 6H, 2CH_3_), 2.68–2.74 (m, 2H, COCH_2_), 3.50 (qui, *J* = 4.0 Hz, 1H, CH), 3.85–4.02 (m, 2H, NCH_2_), 5.65 (s, 2H, 2CH), 6.92–7.26 (m, 3H, Ar-H), 9.99 (s, 1H, NH), 10.86 (s, 1H, OH); ^13^C-NMR (DMSO-*d_6_*, 101 MHz) δ: 10.9 (CH_3_), 33.6 (CH), 35.3 (CH_2_), 51.1 (NCH_2_), 103.1 (CH), 118.0, 121.9, 126.4, 127.9, 151.8 (Ar-C), 171.7, 172.2 (C=O); HRMS (ESI): *m*/*z* calcd for C_17_H_19_ClN_3_O_3_ 348.1115 [M + H]^+^, found 348.1117.

*1-(5-Chloro-2-hydroxyphenyl)-4-(3,5-dimethyl-1H-pyrazole-1-carbonyl)pyrrolidin-2-one* (**13**): *Method A.* To 1-(5-chloro-2-hydroxyphenyl)-5-oxopyrrolidine-3-carbohydrazide (**9**) (1.08 g, 4 mmol) dissolved in hot propan-2-ol (25 mL), pentane-2,4-dione (0.82 mL, 8 mmol) and HCl (2-3 drops) were added. A mixture was heated at reflux for 5 h. Liquid fractions were removed under vacuo, the residue was poured over with water (100 mL), heated to the boiling point until complete dissolution. Precipitate formed after cooling down was filtered off, washed with water, and recrystallized from propan-2-ol. *Method B.* A mixture of 1-(5-chloro-2-hydroxyphenyl)-5-oxopyrrolidine-3-carbohydrazide (**9**) (0.20 g, 0.75 mmol), propan-2-ol (5 mL), and pentane-2,4-dione (0.15 mL, 1.5 mmol) was subjected to microwave irradiation at 100 W at 100 °C for 20 min. Liquid fractions were removed under vacuo, the residue was diluted with water (30 mL), and heated to the boiling point. Precipitate formed after cooling down was filtered off, washed with water, and recrystallized from propan-2-ol. Yield *A* 82%; *B* 60%; white solid; m.p. 99–100 °C; IR (KBr) ν_max_ (cm^−1^): 3130 (OH), 1724, 1662 (C=O); ^1^H-NMR (DMSO-*d_6_*, 400 MHz) δ: 1.20, 1.21 (2s, 6H, 2CH_3_), 2.55–2.71 (m, 2H, COCH_2_), 3.78–3.94 (m, 3H, CH+NCH_2_), 4.90–5.00 (m, 1H, CH), 6.90–7.21 (m, 3H, Ar-H), 10.00 (s, 1H, OH); ^13^C-NMR (DMSO-*d_6_*, 101 MHz) δ: 33.4 (CH), 36.0 (CH_2_), 50.4 (NCH_2_), 52.1 (CH_3_), 108.7, 118.0, 121.9, 126.4, 127.9, 144.1, 151.4, 151.8 (Ar-C), 172.0, 173.1 (C=O); HRMS (ESI): *m*/*z* calcd for C_16_H_17_ClN_3_O_3_ 334.0958 [M + H]^+^, found 334.0521.

#### 3.2.1. General Procedure for Synthesis of **14**–**17**

To 1-(5-chloro-2-hydroxyphenyl)-5-oxopyrrolidine-3-carbohydrazide (**9**) (1.08 g, 4 mmol) dissolved in methanol (25 mL), corresponding cyanate (6 mmol) was added and a reaction mixture was heated at reflux for 10 min – 4 h. Precipitate was filtered off and recrystallized from methanol.

*2-(1-(5-chloro-2-hydroxyphenyl)-5-oxopyrrolidine-3-carbonyl)-N-phenylhydrazine-1-carboxamide* (**14**): Prepared from phenyl isocyanate (0.66 mL, 6 mol) by heating a reaction mixture at reflux for 2 h. Yield 91%; white solid; m.p. 139–140 °C; IR (KBr) ν_max_ (cm^−1^): 3273 (OH), 3040, 2984, 2966 (NH), 1691, 1671, 1642 (C=O); ^1^H-NMR (DMSO-*d_6_*, 300 MHz) δ: 2.64–2.67 (m, 2H, COCH_2_), 3.34–3.40 (m, 1H, CH), 3.72–3.96 (m, 2H, NCH_2_), 6.89–7.47 (m, 8H, Ar-H), 8.10 (s, 1H, NH), 8.78 (s, 1H, NH), 9.91 (s, 1H, OH), 9.95 (s, 1H, NH); ^13^C-NMR (DMSO-*d_6_*, 75 MHz) δ: 33.9 (CH), 35.3 (CH_2_), 51.1 (NCH_2_), 118.0, 118.5, 121.9, 126.6, 127.9, 127.9, 128.7, 139.5, 151.8 (Ar-C), 155.2, 172.3, 172.4 (C=O); HRMS (ESI): *m*/*z* calcd for C_18_H_18_ClN_4_O_4_ 389.1016 [M + H]^+^, found 389.1013.

*2-(1-(5-Chloro-2-hydroxyphenyl)-5-oxopyrrolidine-3-carbonyl)-N-(4-chlorophenyl)hydrazine-1-carboxamide* (**15**): Prepared from 4-chlorophenyl isocyanate (0.77 g, 6 mmol) by heating a reaction mixture at reflux for 10 min. Yield 93%; white solid; m.p. 199–200 °C; IR (KBr) ν_max_ (cm^−1^): 3273 (OH), 3040, 2984, 2966 (NH), 1691, 1671, 1642 (C=O); ^1^H-NMR (DMSO-*d_6_*, 300 MHz) δ: 2.60–2.67 (m, 2H, COCH_2_), 3.34–3.40 (m, 1H, CH), 3.73–3.96 (m, 2H, NCH_2_), 6.91–7.52 (m, 7H, Ar-H), 8.19 (s, 1H, NH), 8.94 (s, 1H, NH), 9.92 (s, 1H, OH), 9.95 (s, 1H, NH); ^13^C-NMR (DMSO-*d_6_*, 75 MHz) δ: 33.9 (CH), 35.3 (CH_2_), 51.7 (NCH_2_), 118.0, 119.6, 120.0, 121.9, 126.6, 127.9, 127.9, 128.6, 138.2, 138.6, 151.7 (Ar-C), 153.9, 172.3, 172.4 (C=O); HRMS (ESI): *m*/*z* calcd for C_18_H_17_Cl_2_N_4_O_4_ 423.0627 [M + H]^+^, found 423.0619.

*2-(1-(5-Chloro-2-hydroxyphenyl)-5-oxopyrrolidine-3-carbonyl)-N-phenylhydrazine-1-carbothioamide* (**16**): Prepared from phenyl isothiocyanate (0.72 mL, 6 mmol) by heating a reaction mixture at reflux for 3 h. Yield 88%; white solid; m.p. 138–139 °C; IR (KBr) ν_max_ (cm^−1^): 3174 (OH), 3018, 2971, 2883 (NH), 1677, 1662 (C=O), 1206 (C=S); ^1^H-NMR (DMSO-*d_6_*, 300 MHz) δ: 2.62–2.70 (m, 2H, COCH_2_), 3.79–4.01 (m, 3H, CH+NCH_2_), 6.93–7.46 (m, 8H, Ar-H), 9.60 (s, 1H, NH), 10.01(s, 1H, OH), 10.19, 11.14 (2s, 2H, 2NH); ^13^C-NMR (DMSO-*d_6_*, 75 MHz) δ: 33.9 (CH), 36.2 (CH_2_), 51.1 (NCH_2_), 118.1, 121.9, 126.6, 127.9, 128.0, 128.9, 130.0, 139.1, 151.8 (Ar-C), 172.2, 172.4 (C=O), 182.5 (C=S); HRMS (ESI): *m*/*z* calcd for C_18_H_18_ClN_4_O_3_S 405.0788 [M + H]^+^, found 405.0784.

*2-(1-(5-Chloro-2-hydroxyphenyl)-5-oxopyrrolidine-3-carbonyl)-N-methylhydrazine-1-carbothioamide* (**17**): Prepared from methyl isothiocyanate (0.44 g, 0.41 mL, 6 mmol) by heating a reaction mixture at reflux for 4 h. Yield 71%; white solid; m.p. 249–250 °C; IR (KBr) ν_max_ (cm^−1^): 3172 (OH), 3056, 2972, 2782 (NH), 1652, 1494 (C=O), 1255 (C=S); ^1^H-NMR (DMSO-*d_6_*, 300 MHz) δ: 2.70–2.91 (m, 2H, COCH_2_), 3.45 (s, 3H, CH_3_), 3.90–4.11 (m, 3H, CH+NCH_2_), 6.92–7.26 (m, 3H, Ar-H), 8.18 (s, 1H, NH), 9.66 (s, 1H, NH), 10.01 (s, 1H, OH), 10.24 (s, 1H, NH); ^13^C-NMR (DMSO-*d_6_*, 75 MHz) δ: 28.8 (CH_3_), 30.0 (CH), 34.1 (CH_2_); 51.1 (NCH_2_), 118.1, 121.9, 126.5, 128.0, 151.8 (Ar-C), 152.9 (C=O), 167.4 (C=S), 172.0 (C=O); HRMS (ESI): *m*/*z* calcd for C_13_H_16_ClN_4_O_3_S 343.0631 [M + H]^+^, found 343.0740.

*1-(5-Chloro-2-hydroxyphenyl)-4-(5-(methylamino)-1,3,4-thiadiazol-2-yl)pyrrolidin-2-one* (**18**): To conc. H_2_SO_4_ (5 mL) carbothioamide **17** (0.68 g, 2 mmol) was added in portions and a reaction mixture was stirred at room temperature for 3 h. Afterwards, it was added dropwise into water-ice mixture. Precipitate was filtered off and recrystallized from methanol. Yield 62%; white solid; m.p. 249–250 °C; IR (KBr) ν_max_ (cm^−1^): 3172 (OH), 3055, 2970 (NH), 1652 (C=O); ^1^H-NMR (DMSO-*d_6_*, 300 MHz) δ: 2.72–2.90 (m, 2H, COCH_2_), 3.45 (s, 3H, CH_3_), 3.85–4.15 (m, 3H, CH+NCH_2_), 6.88–7.30 (m, 3H, Ar-H), 9.97 (s, 1H, OH), 13.64 (s, 1H, NH); ^13^C-NMR (DMSO-*d_6_*, 75 MHz) δ: 28.8 (CH_3_), 29.9 (CH), 34.1 (CH_2_), 51.1 (NCH_2_), 118.1, 121.9, 126.4, 127.9, 128.0, 151.8, 152.9, 167.4 (Ar-C), 171.9 (C=O); HRMS (ESI): *m*/*z* calcd for C_13_H_14_ClN_4_O_2_S 325.0526 [M + H]^+^, found 325.0519.

#### 3.2.2. General Procedure for Synthesis of **19**–**21**

A mixture of a corresponding carboxamide (2 mmol) and aqueous 10% KOH solution (25 mL) was heated at reflux for 12 h. After cooling down, 10% HCl solution was added to pH 5. Precipitate was filtered off and recrystallized from DMF/H_2_O mixture. 

*5-(1-(5-Chloro-2-hydroxyphenyl)-5-oxopyrrolidin-3-yl)-4-phenyl-2,4-dihydro-3H-1,2,4-triazol-3-one* (**19**): Prepared from carboxamide **14** (0.78 g, 2 mmol). Yield 71%; white solid; m.p. 124–125 °C; IR (KBr) ν_max_ (cm^−1^): 3195 (OH), 1704, 1695 (C=O); ^1^H-NMR (DMSO-*d_6_*, 300 MHz) δ: 2.65–2.93 (m, 2H, COCH_2_), 3.85–4.14 (m, 3H, CH+NCH_2_), 6.86–7.30 (m, 8H, Ar-H), 9.99 (s, 1H, OH), 13.63 (s, 1H, NH); ^13^C-NMR (DMSO-*d_6_*, 75 MHz) δ: 33.9 (CH), 34.4 (CH_2_), 50.9 (NCH_2_), 118.0, 121.8, 126.3, 127.8, 128.8, 132.6, 137.8, 142.8, 151.7, 154.4 (Ar-C), 171.8, 172.9 (C=O); HRMS (ESI): *m*/*z* calcd for C_18_H_16_ClN_4_O_3_S 371.0911 [M + H]^+^, found 371.0907.

*1-(5-Chloro-2-hydroxyphenyl)-4-(4-phenyl-5-thioxo-4,5-dihydro-1H-1,2,4-triazol-3-yl)pyrrolidin-2-one* (**20**): Prepared from carbothioamide **16** (0.81 g, 2 mmol). Yield 90%; white solid; m.p. 266–267 °C; IR (KBr) ν_max_ (cm^−1^): 3069 (OH), 3017 (NH), 1665 (C=O), 1276 (C=S); ^1^H-NMR (DMSO-*d_6_*, 300 MHz) δ: 2.69–2.78 (m, 2H, COCH_2_), 3.57–3.65 (m, 2H, NCH_2_), 3.80–3.88 (m, 1H, CH), 6.87–7.64 (m, 8H, Ar-H), 9.94 (s, 1H, OH), 13.88 (s, 1H, NH); ^13^C-NMR (DMSO-*d_6_*, 75 MHz) δ: 29.1 (CH), 34.4 (CH_2_), 51.1 (NCH_2_), 118.0, 121.9, 126.2, 128.0, 128.6, 129.7, 129.8, 133.6, 151.8, 152.7 (Ar-C), 168.4 (C=O), 171.7 (C=S); HRMS (ESI): *m*/*z* calcd for C_18_H_16_ClN_4_O_2_S 387.0678 [M + H]^+^, found 387.0679.

*1-(5-Chloro-2-hydroxyphenyl)-4-(4-methyl-5-thioxo-4,5-dihydro-1H-1,2,4-triazol-3-yl)pyrrolidin-2-one* (**21**): Prepared from carbothioamide **17** (0.68 g, 2 mmol). Yield 72%; white solid; m.p. 244–245 °C; IR (KBr) ν_max_ (cm^−1^): 3169 (OH), 2971 (NH), 1652 (C=O), 1254 (C=S); ^1^H-NMR (DMSO-*d_6_*, 300 MHz) δ: 2.65–2.91 (m, 2H, COCH_2_), 3.46 (s, 3H, CH_3_), 3.80–4.14 (m, 3H, CH+NCH_2_), 6.91–7.24 (m, 3H, Ar-H), 9.98 (s, 1H, OH), 13.64 (s, 1H, NH); ^13^C-NMR (DMSO-*d_6_*, 75 MHz) δ: 28.8 (CH_3_), 29.9 (CH), 34.1 (CH_2_), 51.1 (NCH_2_), 118.1, 121.9, 126.4, 127.9, 151.8, 152.9 (Ar-C), 167.4 (C=O), 171.9 (C=S); HRMS (ESI): *m*/*z* calcd for C_13_H_14_ClN_4_O_2_S 325.0526 [M + H]^+^, found 325.0523.

#### 3.2.3. General procedure for synthesis of **22**–**26**

To 1-(5-chloro-2-hydroxyphenyl)-4-(4-phenyl-5-thioxo-4,5-dihydro-1*H*-1,2,4-triazol-3-yl)pyrrolidin-2-one (**20**) (0.77 g, 2 mmol) dissolved in DMF (5 mL), triethylamine (0.51 g, 0.70 mL, 5 mmol) and haloalkane (3 mmol) were added. The reaction mixture was stirred at room temperature for 4 h and then diluted with cold water (30 mL). Precipitate was filtered off and recrystallized from propan-2-ol (unless indicated otherwise).

*1-(5-Chloro-2-hydroxyphenyl)-4-(5-(ethylthio)-4-phenyl-4H-1,2,4-triazol-3-yl)pyrrolidin-2-one* (**22**): Prepared from iodoethane (0.47g, 0.24 mL, 3 mmol). Yield 69%; white solid; m.p. 161–162 °C; IR (KBr) ν_max_ (cm^−1^): 3061 (OH), 1695 (C=O); ^1^H-NMR (DMSO-*d_6_*, 300 MHz) δ: 1.29 (t, *J* = 7.2 Hz, 3H, CH_3_), 2.52–2.59 (m, 1H, COCH_2_); 2.72–2.81 (m, 1H, CH_2_); 3.08 (q, *J* = 7.2 Hz, 2H, CH_3_*CH_2_*), 3.63–3.98 (m, 2H, CH+NCH_2_), 6.89–7.65 (m, 8H, Ar-H), 10.12 (s, 1H, OH); ^13^C-NMR (DMSO-*d_6_*, 75 MHz) δ: 14.8 (CH_3_), 26.5 (*C*H_2_CH_3_), 28.8 (CH), 35.3 (CH_2_), 52.0 (NCH_2_), 118.1, 121.8, 126.3, 127.6, 128.1, 130.0, 130.2, 132.8, 150.9, 152.0, 156.4 (Ar-C), 171.9 (C=O); HRMS (ESI): *m*/*z* calcd for C_20_H_20_ClN_4_O_2_S 415.0995 [M + H]^+^, found 415.0999.

*Ethyl 2-((5-(1-(5-chloro-2-hydroxyphenyl)-5-oxopyrrolidin-3-yl)-4-phenyl-4H-1,2,4-triazol-3-yl)thio)acetate* (**23**): Prepared from ethyl chloroacetate (0.37 g, 0.32 mL, 3 mmol). Yield 82%; white solid; m.p. 174–175 °C; IR (KBr) ν_max_ (cm^−1^): 3068 (OH), 1749, 1670 (C=O); ^1^H-NMR (DMSO-*d_6_*, 300 MHz) δ: 1.18 (t, *J* = 7.2 Hz, 3H, *CH_3_*CH_2_), 2.53–2.78 (m, 2H, COCH_2_), 3.35 (s, 1H, CH), 3.65–3.80 (m, 2H, NCH_2_), 3.89–3.97 (m, 1H, CH), 4.11 (q, *J* = 7.2 Hz, *J* = 14.1 Hz, 2H, CH_3_*CH_2_*), 4.06 (s, 2H, CH_2_), 4.09–4.17 (m, 2H, CH_2_), 6.87–7.68 (m, 8H, Ar-H), 10.07 (s, 1H, OH); ^13^C-NMR (DMSO-*d_6_*, 75 MHz) δ: 14.0 (CH_3_), 28.8 (*C*H_2_CH_3_), 33.9 (CH), 35.3 (CH_2_), 52.0 (NCH_2_), 61.3 (CH_3_*CH_2_*), 118.1, 121.8, 126.3, 127.5, 128.0, 130.1, 130.4, 132.5, 150.2, 151.9, 156.5 (Ar-C), 168.0, 171.8 (C=O); HRMS (ESI): *m*/*z* calcd for C_22_H_22_ClN_4_O_4_S 473.1050 [M + H]^+^, found 473.1054.

*1-(5-Chloro-2-hydroxyphenyl)-4-(5-((2-oxo-2-phenylethyl)thio)-4-phenyl-4H-1,2,4-triazol-3-yl)pyrrolidin-2-one* (**24**): Prepared from 2-bromo-1-phenylethanone (0.60 g, 3 mmol). Yield 87%; white solid; m.p. 99–100 °C (propan-2-ol/H_2_O); IR (KBr) ν_max_ (cm^−1^): 3063 (OH), 1695, 1680 (C=O); ^1^H-NMR (DMSO-*d_6_*, 300 MHz) δ: 2.53–2.78 (m, 2H, COCH_2_), 3.35 (s, 1H, CH), 3.58–3.96 (m, 2H, NCH_2_), 4.89 (s, 2H, CH_2_), 6.87–8.02 (m, 13H, Ar-H), 10.08 (s, 1H, OH); ^13^C-NMR (DMSO-*d_6_*, 75 MHz) δ: 28.8 (CH), 35.3 (CH_2_), 40.1 (*C*H_2_CO), 52.0 (NCH_2_), 118.1, 121.8, 126.2, 127.5, 128.0, 128.4, 128.8, 130.1, 130.4, 132.6, 133.7, 135.3, 150.5, 151.9, 156.4 (Ar-C), 171.8, 193.0 (C=O); HRMS (ESI): *m*/*z* calcd for C_26_H_22_ClN_4_O_3_S 505.1101 [M + H]^+^, found 505.1095.

*1-(5-Chloro-2-hydroxyphenyl)-4-(5-((2-(4-chlorophenyl)-2-oxoethyl)thio)-4-phenyl-4H-1,2,4-triazol-3-yl)pyrrolidin-2-one* (**25**): Prepared from 2-bromo-1-(4-chlorophenyl)ethan-1-one (0.70 g, 3 mmol). Yield 83%; white solid; m.p. 69–70 °C; IR (KBr) ν_max_ (cm^−1^): 3064 (OH), 1699, 1678 (C=O); ^1^H-NMR (DMSO-*d_6_*, 300 MHz) δ: 2.53–2.77 (m, 2H, COCH_2_), 3.65–3.79 (m, 2H, NCH_2_), 3.87–3.95 (m, 1H, CH), 4.87 (s, 2H, CH_2_), 6.84–8.07 (m, 12H, Ar-H), 10.04 (s, 1H, OH); ^13^C-NMR (DMSO-*d_6_*, 75 MHz) δ: 28.8 (CH), 35.3 (CH_2_), 40.0 (*C*H_2_CO), 52.0 (NCH_2_), 118.1, 121.8, 126.2, 127.5, 128.0, 128.9, 130.1, 130.3, 130.6, 132.5, 134.0, 138.7, 150.4, 151.9, 156.5 (Ar-C), 171.8, 192.2 (C=O); HRMS (ESI): *m*/*z* calcd for C_26_H_21_Cl_2_N_4_O_3_S 539.0711 [M + H]^+^, found 539.0706.

*1-(5-Chloro-2-hydroxyphenyl)-4-(5-((2-(4-nitrophenyl)-2-oxoethyl)thio)-4-phenyl-4H-1,2,4-triazol-3-yl)pyrrolidin-2-one* (**26**): Prepared from 2-bromo-1-(4-nitrophenyl)ethanone (0.73 g, 3 mmol). Yield 81%; white solid; m.p. 104–105 °C; IR (KBr) ν_max_ (cm^−1^): 3071 (OH), 1703, 1601 (C=O); ^1^H-NMR (DMSO-*d_6_*, 300 MHz) δ: 2.41–2.48 (m, 1H, COCH_2_), 2.69–2.78 (m, 2H, CH+COCH_2_), 3.57–3.66 (m, 2H, NCH_2_), 3.80–3.88 (m, 2H, CH_2_), 6.84–7.63 (m, 12H, Ar-H), 9.91 (s, 1H, OH); ^13^C-NMR (DMSO-*d_6_*, 75 MHz) δ: 29.1 (CH), 34.4 (CH_2_), 39.9 (*C*H_2_CO), 51.0 (NCH_2_), 118.0, 121.8, 126.2, 127.9, 128.5, 129.6, 129.7, 133.55, 151.7, 152.6 (Ar-C), 168.4, 171.6 (C=O); HRMS (ESI): *m*/*z* calcd for C_26_H_21_ClN_5_O_5_S 550.0952 [M + H]^+^, found 550.0945.

### 3.3. X-ray Diffraction Study of Compound **11**

Diffraction data for **11** were collected at −100 °C on a Bruker-Nonius KappaCCD diffractometer (Bruker AXS B.V., Delft, The Netherlands) using graphite monochromated Mo-Kα radiation (λ = 0.71073 Å). The crystal structure of **11** was solved by direct methods [28] and refined by full-matrix least squares [29]. All nonhydrogen atoms were refined in anisotropical approximation. Hydrogen atoms involved in H-bonds were refined in isotropical approximation; hydrogen atoms bonded to carbon atoms were refined by riding model with *U*iso(H) = 1.2*U*eq(C). Crystal data for **11**: monoclinic: *a* = 12.5834(4), *b* = 4.6576(2), *c* = 15.5408(6) Å, *β* = 94.626(2)°; *V* = 907.86(6) Å^3^, *Z* = 2, *μ* = 0.248 mm^–1^, *D*calc = 1.462 g·cm^–3^; space group is *Pa*. For further details, see crystallographic data for **11** deposited at the Cambridge Crystallographic Data Centre as Supplementary Publication Number CCDC 1883850. Copies of the data can be obtained, free of charge, on application to CCDC, 12 Union Road, Cambridge CB2 1EZ, UK.

### 3.4. Screening of Antioxidant Properties

#### 3.4.1. DPPH (1,1-diphenyl-2-picrylhydrazyl) Radical Scavenging Assay

The free radical scavenging activity of compounds was measured with DPPH using the widely used method [30]. Briefly, a 1 mM solution of DPPH in ethanol was prepared, and this solution (1 mL) was added to the solutions of the tested compounds (1 mg/mL of DMSO). The mixture was shaken vigorously and allowed to stand at room temperature for 20 min. Afterwards, the absorbance was measured at 517 nm with a UV-200-RS spectrophotometer (MRC Ltd., Holon, Israel). The lower absorbance of the reaction mixture indicated a higher free radical scavenging activity. The capability to scavenge the DPPH radical was calculated according to the following equation:

DPPH scavenging effect (%) = (A_0_ – A_1_/A_0_) × 100

where A_0_ is the absorbance of the control reaction and A_1_ is the absorbance in the presence of the compounds.

Each experiment was repeated three times.

#### 3.4.2. Reducing Power Assay

The solutions of the tested compounds (1000 µg/mL) were mixed with the phosphate buffer (2.5 mL, 0.2 mol/L, pH 6.6) and potassium ferricyanide [K_3_Fe(CN)_6_] (2.5 mL, 1%). The mixture was incubated at 50 °C for 20 min and 10% trichloroacetic acid was added to the mixture, which was then centrifuged at 3000 rpm for 10 min. The upper layer of the solution was diluted with distilled water (2.5 mL), FeCl_3_ (0.5 mL, 0.1%) was added, and the absorbance was measured at 700 nm. The increased absorbance of the reaction mixture indicated an increased reducing power [31]. Each experiment was repeated three times.

## 4. Conclusions

A series of 1-(5-chloro-2-hydroxyphenyl)-5-oxopyrrolidine-3-carboxylic acid derivatives containing chloro, hydroxyl, isopropyl, nitroso, and amino substituents at benzene ring and bearing acyclic and heterocyclic substituents at position 3 of the pyrrolidine ring were synthesized. The results of the antioxidant activity of the synthesized compounds by DPPH radical scavenging method and reducing power assay have revealed that a number of compounds are potent antioxidants. Antioxidant activity of 1-(5-chloro-2-hydroxyphenyl)-4-(5-thioxo-4,5-dihydro-1,3,4-oxadiazol-2-yl)pyrrolidin-2-one has been tested to be 1.5 times higher than that of a well-known antioxidant ascorbic acid. 1-(5-Chloro-2-hydroxyphenyl)-4-(4-methyl-5-thioxo-4,5-dihydro-1*H*-1,2,4-triazol-3-yl)pyrrolidin-2-one has shown 1.35 times higher antioxidant activity in comparison with that of vitamin C by DPPH radical scavenging method and optical density value of 1.149 in reducing power assay.

## Figures and Tables

**Table 1:** Antioxidant activity of compounds **1**–**26** evaluated by DPPH radical scavenging method and reducing power assay.

Compound	Antioxidant Activity According to	
	DPPH Radical Scavenging Method, %	Reducing Power Assay, Optical Density
**1**	22.2	0.094
**2**	24.3	0.107
**3**	22.2	0.094
**4**	20.6	0.117
**5**	41.3	0.278
**6**	55.7	1.675
**7**	51.5	1.573
**8**	42.3	0.053
**9**	46.8	0.316
**10**	88.6	0.446
**11**	49.9	0.011
**12**	49.2	0.299
**13**	16.7	0.071
**14**	53.8	0.191
**15**	20.8	1.346
**16**	50.3	0.574
**17**	50.0	0.223
**18**	47.9	0.249
**19**	87.7	0.623
**20**	58.4	0.303
**21**	78.6	1.149
**22**	48.0	0.024
**23**	30.0	0.456
**24**	53.9	0.097
**25**	59.1	0.128
**26**	66.8	0.115
**Control (ascorbic acid)**	58.2	2.39

## Supplementary Materials

The following are available online at https://www.mdpi.com/1420-3049/24/5/971/s1, Figures S1–S77 display ^1^H-NMR, ^13^C-NMR, and HRMS spectra of compounds **1**–**26**.
