# Supplementary material for: Synthesis of 1-(5-Chloro-2-hydroxyphenyl)-5-oxopyrrolidine-3-carboxylic Acid Derivatives and Their Antioxidant Activity
Source: Molecules. 2019 Mar 9;24(5):971. doi: 10.3390/molecules24050971 (PMC6429199; doi:10.3390/molecules24050971)
Supplement: Supplementary file 1 [file molecules-24-00971-s001.pdf]

Supplementary Materials

Synthesis of 1-(5-Chloro-2-hydroxyphenyl)-5-oxopyrrolidine-3-carboxylic Acid Derivatives and Their Antioxidant Activity

Ingrida Tumosienė [1], Kristina Kantminienė [2,*], Ilona Jonuškienė [1], Artūras Peleckis [1], Sergey Belyakov [3] and Vytautas Mickevičius [1]

[1] Department of Organic Chemistry, Kaunas University of Technology, LT-50254, Kaunas, Lithuania; vytautas.mickevicius@ktu.lt

[2] Department of Physical and Inorganic Chemistry, Kaunas University of Technology, LT-50254, Kaunas, Lithuania; kristina.kantminiene@ktu.lt

[3] Laboratory of Physical Organic Chemistry, Latvian Institute of Organic Synthesis, Aizkraukles 21, 1006 Riga, Latvia; serg@osi.lv

Table of contents

Figure S1. ^{1}H-NMR (400 MHz, DMSO-d$_6$) spectrum of **1**. S5

Figure S2. ^{13}C-NMR (400 MHz, DMSO-d$_6$) spectrum of **1**.

Figure S3. HRMS spectrum of **1**.

Figure S4. ^{1}H-NMR (400 MHz, DMSO-d$_6$) spectrum of **2**. S6

Figure S5. ^{13}C-NMR (400 MHz, DMSO-d$_6$) spectrum of **2**.

Figure S6. HRMS spectrum of **2**.

Figure S7. ^{1}H-NMR (400 MHz, DMSO-d$_6$) spectrum of **3**. S7

Figure S8. ^{13}C-NMR (400 MHz, DMSO-d$_6$) spectrum of **3**.

Figure S9. HRMS spectrum of **3**.

Figure S10. ^{1}H-NMR (400 MHz, DMSO-d$_6$) spectrum of **4**. S8

Figure S11. ^{13}C-NMR (400 MHz, DMSO-d$_6$) spectrum of **4**.

Figure S12. HRMS spectrum of **4**.

Figure S13. ^{1}H-NMR (400 MHz, DMSO-d$_6$) spectrum of **5**. S9

Figure **S14**. ^{13}C-NMR (400 MHz, DMSO-d$_6$) spectrum of **5**.

Figure S15. HRMS spectrum of **5**.
Figure S16. ^{1}H-NMR (400 MHz, DMSO-d_6) spectrum of **6**. S10
Figure S17. ^{13}C-NMR (400 MHz, DMSO-d_6) spectrum of **6**.
Figure S18. HRMS spectrum of **6**.
Figure S19. ^{1}H-NMR (400 MHz, DMSO-d_6) spectrum of **7**. S11
Figure S20. ^{13}C-NMR (400 MHz, DMSO-d_6) spectrum of **7**.
Figure S21. HRMS spectrum of **7**.
Figure S22. ^{1}H-NMR (400 MHz, DMSO-d_6) spectrum of **8**. S12
Figure S23. ^{13}C-NMR (400 MHz, DMSO-d_6) spectrum of **8**.
Figure S24. HRMS spectrum of **8**.
Figure S25. ^{1}H-NMR (400 MHz, DMSO-d_6) spectrum of **9**. S13
Figure S26. ^{13}C-NMR (400 MHz, DMSO-d_6) spectrum of **9**.
Figure S27. HRMS spectrum of **9**.
Figure S28. ^{1}H-NMR (400 MHz, DMSO-d_6) spectrum of **10**. S14
Figure S29. ^{13}C-NMR (400 MHz, DMSO-d_6) spectrum of **10**.
Figure S30. ^{1}H-NMR (400 MHz, DMSO-d_6) spectrum of **11**. S15
Figure S31. ^{13}C-NMR (400 MHz, DMSO-d_6) spectrum of **11**.
Figure S32. HRMS spectrum of **11**.
Figure S33. ^{1}H-NMR (400 MHz, DMSO-d_6) spectrum of **12**. S16
Figure S34. ^{13}C-NMR (400 MHz, DMSO-d_6) spectrum of **12**.
Figure S35. HRMS spectrum of **12**.
Figure S36. ^{1}H-NMR (400 MHz, DMSO-d_6) spectrum of **13**. S17
Figure S37. ^{13}C-NMR (400 MHz, DMSO-d_6) spectrum of **13**.
Figure S38. HRMS spectrum of **13**.
Figure S39. ^{1}H-NMR (400 MHz, DMSO-d_6) spectrum of **14**. S18
Figure S40. ^{13}C-NMR (400 MHz, DMSO-d_6) spectrum of **14**.

Figure S41. HRMS spectrum of **14**.

Figure S42. ^{1}H-NMR (400 MHz, DMSO-d_6) spectrum of **15**. S19

Figure S43. ^{13}C-NMR (400 MHz, DMSO-d_6) spectrum of **15**.

Figure S44. HRMS spectrum of **15**.

Figure S45. ^{1}H-NMR (400 MHz, DMSO-d_6) spectrum of **16**. S20

Figure S46. ^{13}C-NMR (400 MHz, DMSO-d_6) spectrum of **16**.

Figure S47. HRMS spectrum of **16**.

Figure S48. ^{1}H-NMR (400 MHz, DMSO-d_6) spectrum of **17**. S21

Figure S49. ^{13}C-NMR (400 MHz, DMSO-d_6) spectrum of **17**.

Figure S50. HRMS spectrum of **17**.

Figure S51. ^{1}H-NMR (400 MHz, DMSO-d_6) spectrum of **18**. S22

Figure S52. ^{13}C-NMR (400 MHz, DMSO-d_6) spectrum of **18**.

Figure S53. HRMS spectrum of **18**.

Figure S54. ^{1}H-NMR (400 MHz, DMSO-d_6) spectrum of **19**. S23

Figure S55. ^{13}C-NMR (400 MHz, DMSO-d_6) spectrum of **19**.

Figure S56. HRMS spectrum of **19**.

Figure S57. ^{1}H-NMR (400 MHz, DMSO-d_6) spectrum of **20**. S24

Figure S58. ^{13}C-NMR (400 MHz, DMSO-d_6) spectrum of **20**.

Figure S59. HRMS spectrum of **20**.

Figure S60. ^{1}H-NMR (400 MHz, DMSO-d_6) spectrum of **21**. S25

Figure S61. ^{13}C-NMR (400 MHz, DMSO-d_6) spectrum of **21**.

Figure S62. HRMS spectrum of **21**.

Figure S63. ^{1}H-NMR (400 MHz, DMSO-d_6) spectrum of **22**. S26

Figure S64. ^{13}C-NMR (400 MHz, DMSO-d_6) spectrum of **22**.

Figure S65. HRMS spectrum of **22**.

Figure S66. ^{1}H-NMR (400 MHz, DMSO-d_6) spectrum of **23**. S27

Figure S67. ^{13}C-NMR (400 MHz, DMSO-d_6) spectrum of **23**.

Figure S68. HRMS spectrum of **23**.

Figure S69. ^{1}H-NMR (400 MHz, DMSO-d_6) spectrum of **24**. S28

Figure S70. ^{13}C-NMR (400 MHz, DMSO-d_6) spectrum of **24**.

Figure S71. HRMS spectrum of **24**.

Figure S72. ^{1}H-NMR (400 MHz, DMSO-d_6) spectrum of **25**. S29

Figure S73. ^{13}C-NMR (400 MHz, DMSO-d_6) spectrum of **25**.

Figure S74. HRMS spectrum of **25**.

Figure S75. ^{1}H-NMR (400 MHz, DMSO-d_6) spectrum of **26**. S30

Figure S76. ^{13}C-NMR (400 MHz, DMSO-d_6) spectrum of **26**.

Figure S77. HRMS spectrum of **26**.

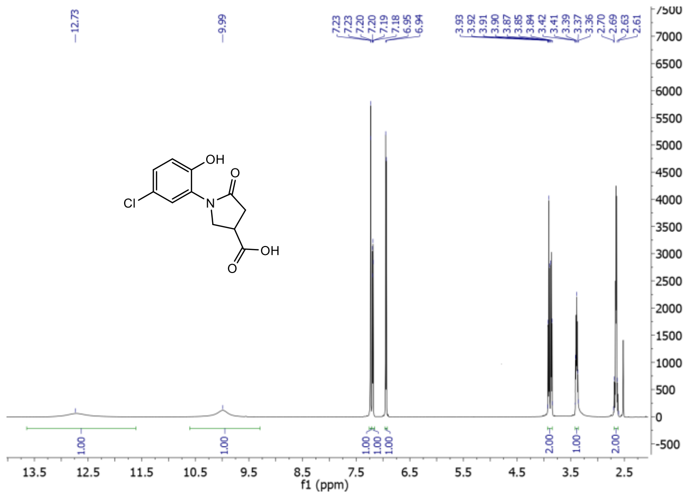

Figure S1. ^{1}H-NMR (400 MHz, DMSO-d_6) spectrum of **1**.

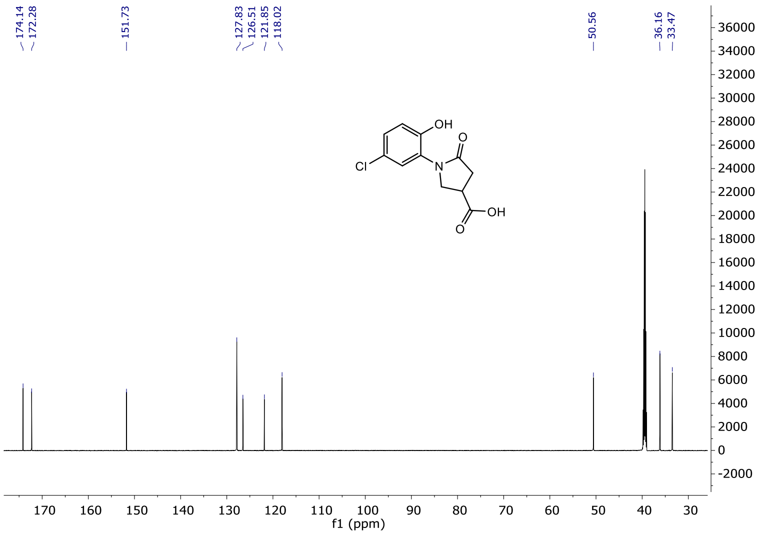

Figure S2. ^{13}C-NMR (400 MHz, DMSO-d_6) spectrum of **1**.

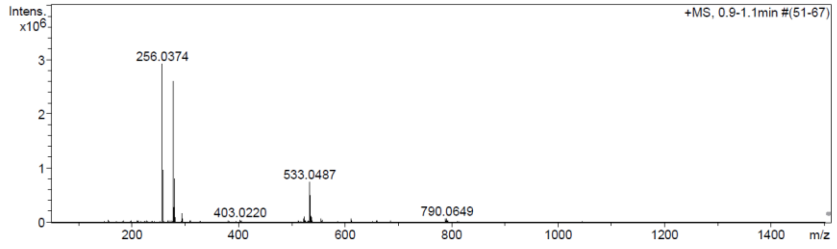

Meas. m/z	#	Formula	Score	m/z	err [ppm]	Mean err [ppm]	mSigma	rdb	e⁻ Conf	N-Rule
256.0374	1	C 11 H 11 Cl N O 4	100.00	256.0371	-1.3	-1.0	10.0	6.5	even	ok

Figure S3. HRMS spectrum of **1**.

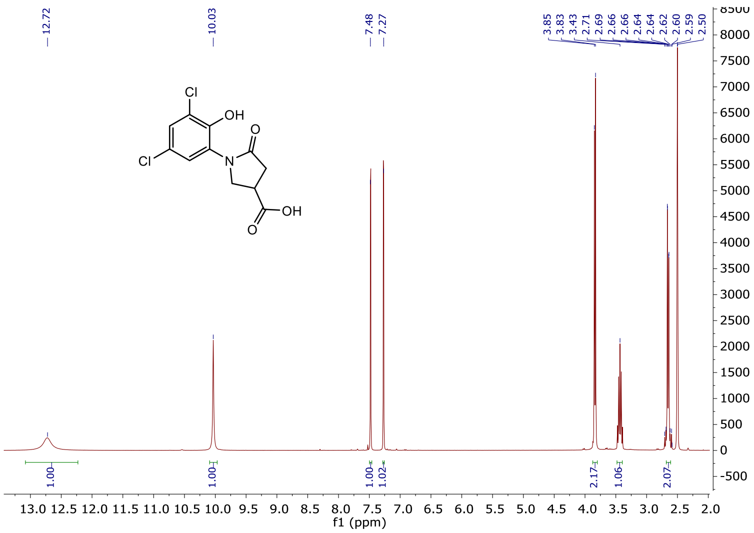

Figure S4. ^{1}H-NMR (400 MHz, DMSO-d_6) spectrum of **2**.

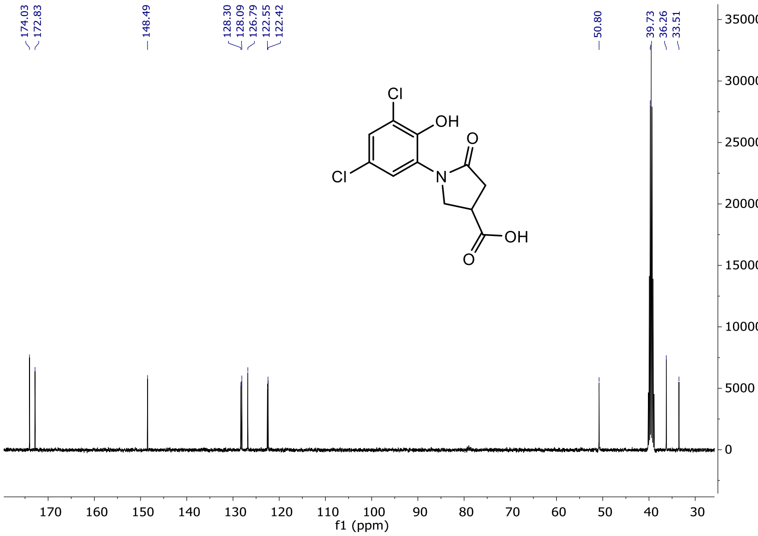

Figure S5. ^{13}C-NMR (400 MHz, DMSO-d_6) spectrum of **2**.

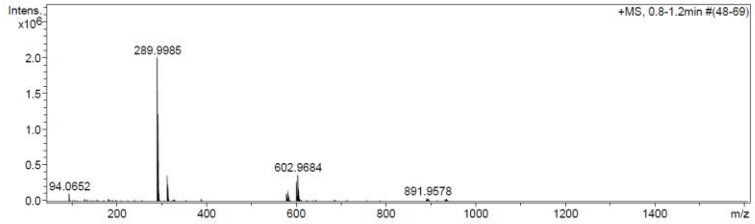

Figure S6. HRMS spectrum of **2**.

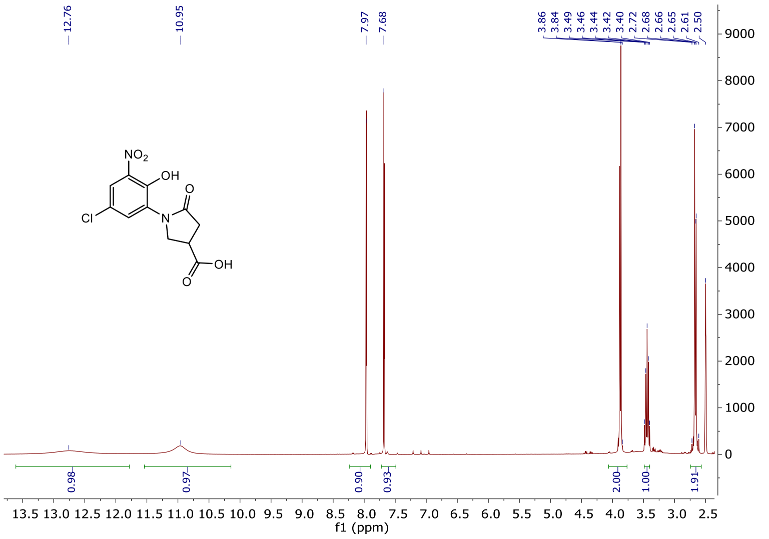

Figure S7. ^{1}H-NMR (400 MHz, DMSO-d$_6$) spectrum of **3**.

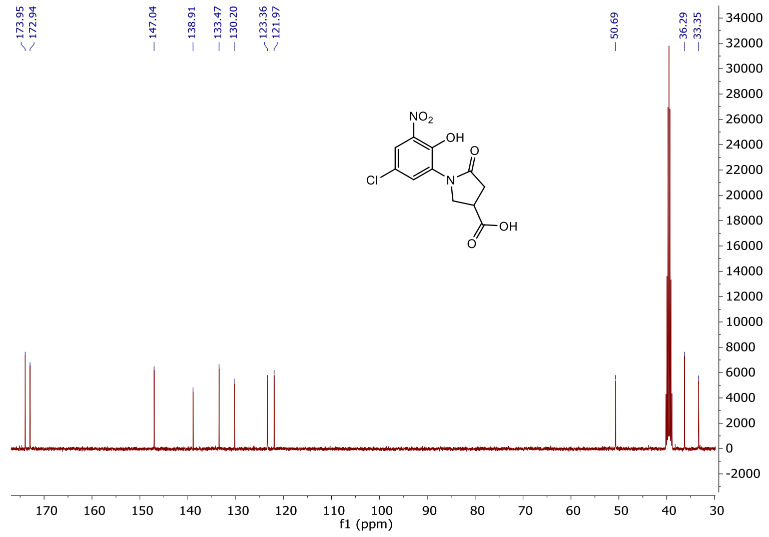

Figure S8. ^{13}C-NMR (400 MHz, DMSO-d$_6$) spectrum of **3**.

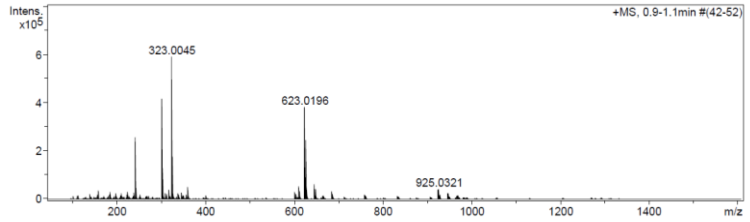

Meas. m/z	#	Formula	Score	m/z	err [ppm]	Mean err [ppm]	mSigma	rdb	e⁻ Conf	N-Rule
242.2843	1	C 16 H 36 N	100.00	242.2842	-0.4	-0.2	3.3	-0.5	even	ok
301.0224	1	C 8 H 2 Cl N 12	51.00	301.0208	-5.2	-5.2	9.3	13.5	even	ok
	2	C 11 H 10 Cl N 2 O 6	100.00	301.0222	-0.7	-0.6	13.1	7.5	even	ok

Figure S9. HRMS spectrum of **3**.

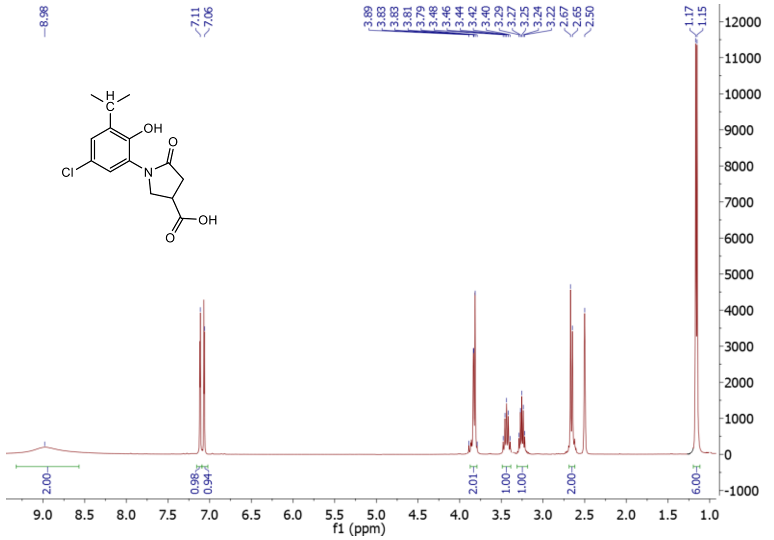

Figure S10. ^{1}H-NMR (400 MHz, DMSO-d_6) spectrum of **4**.

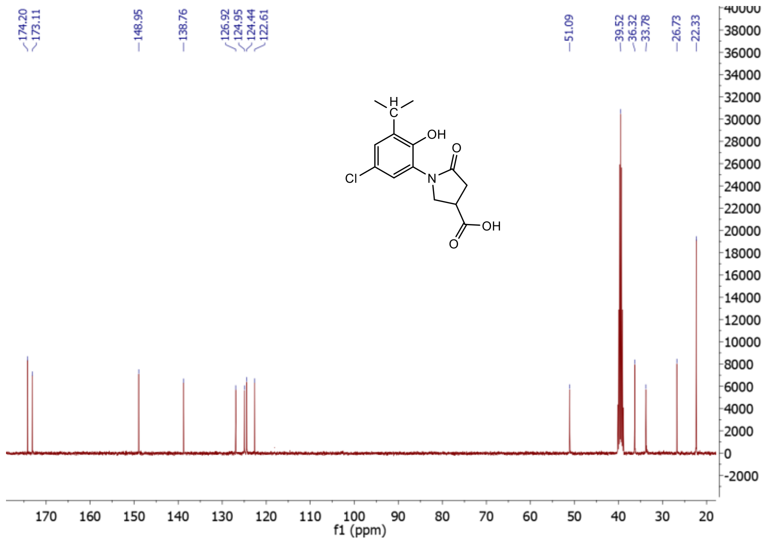

Figure S11. ^{13}C-NMR (400 MHz, DMSO-d_6) spectrum of **4**.

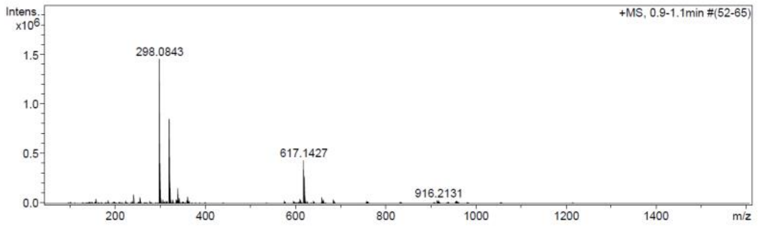

Meas. m/z	#	Formula	Score	m/z	err [ppm]	Mean err [ppm]	mSigma	rdb	e⁻ Conf	N-Rule
298.0843	1	C 14 H 17 Cl N O 4	100.00	298.0841	-0.8	-0.7	33.8	6.5	even	ok

Figure S12. HRMS spectrum of **4**.

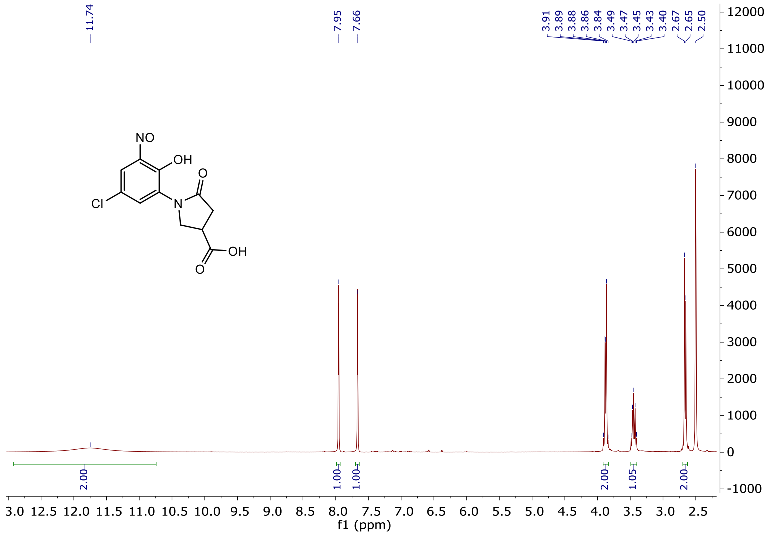

Figure S13. ^{1}H-NMR (400 MHz, DMSO-d$_6$) spectrum of **5**.

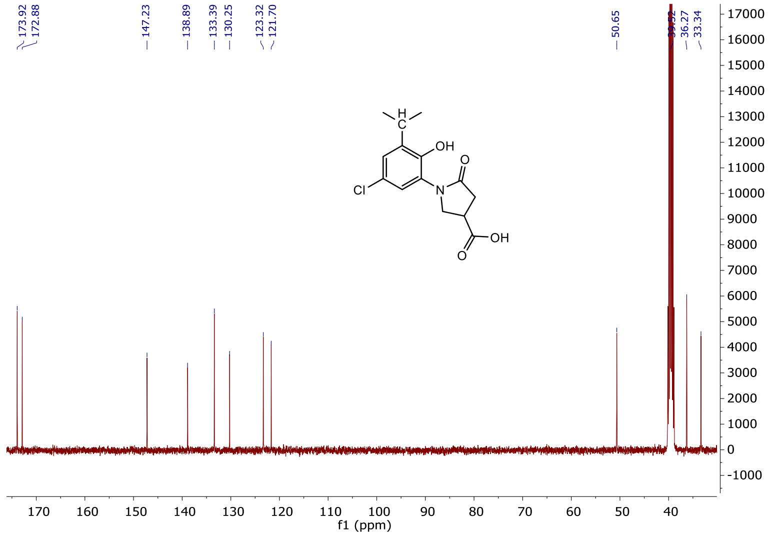

Figure S14. ^{13}C-NMR (400 MHz, DMSO-d$_6$) spectrum of **5**.

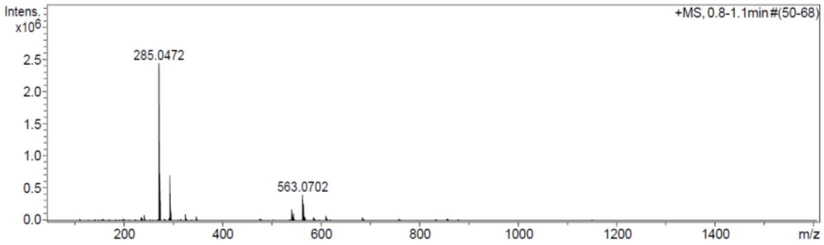

Meas. m/z	#	Formula	Score	m/z	err [ppm]	Mean err [ppm]	mSigma	rdb	e⁻ Conf	N-Rule
285.0472	1	C 11 H 9 Cl N 2 O 5	100.00	285.0461	-0.8	-0.4	21.0	6.5	even	ok

Figure S15. HRMS spectrum of **5**.

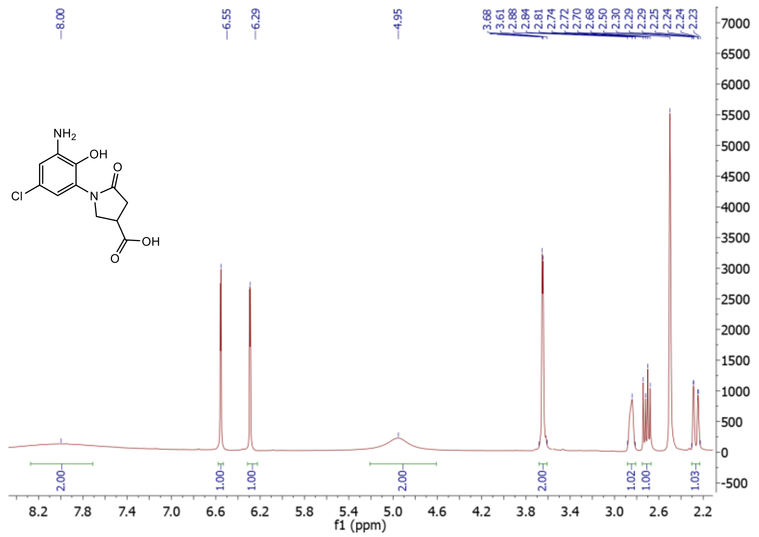

Figure S16. ^{1}H-NMR (400 MHz, DMSO-d6) spectrum of **6**.

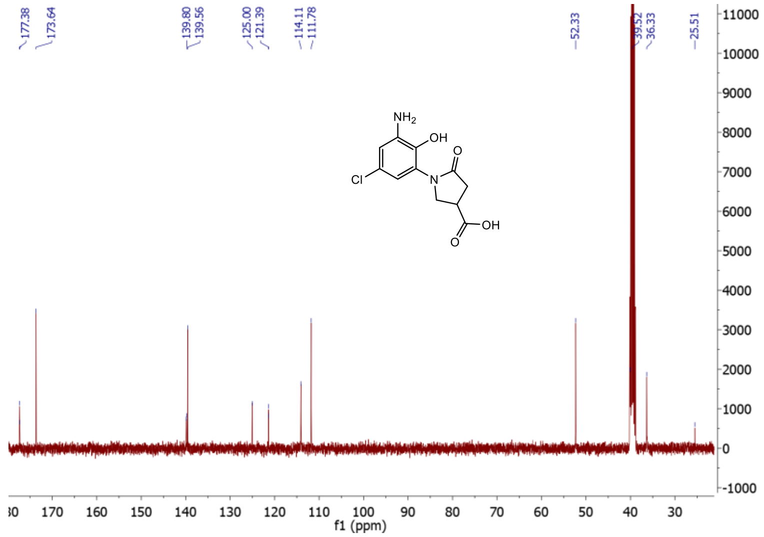

Figure S17. ^{13}C-NMR (400 MHz, DMSO-d6) spectrum of **6**.

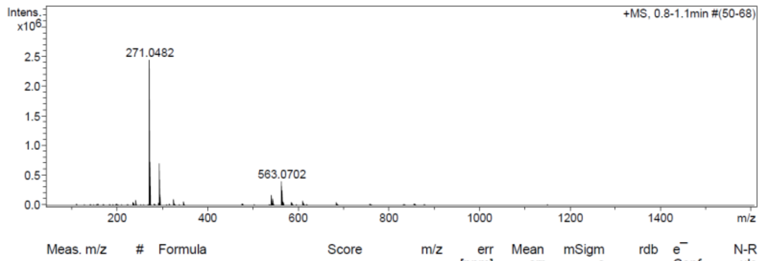

Meas. m/z	#	Formula	Score	m/z	err [ppm]	Mean err [ppm]	mSigma	rdb	e⁻ Conf	N-Rule
271.0482	1	C 11 H 12 Cl N 2 O 4	100.00	271.0480	-0.8	-0.4	21.0	6.5	even	ok

Figure S18. HRMS spectrum of **6**.

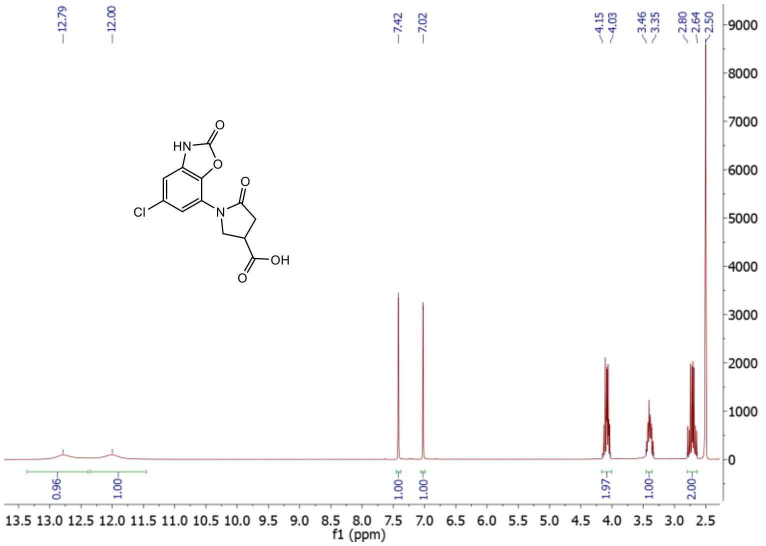

Figure S19. ^{1}H-NMR (400 MHz, DMSO-d_6) spectrum of **7**.

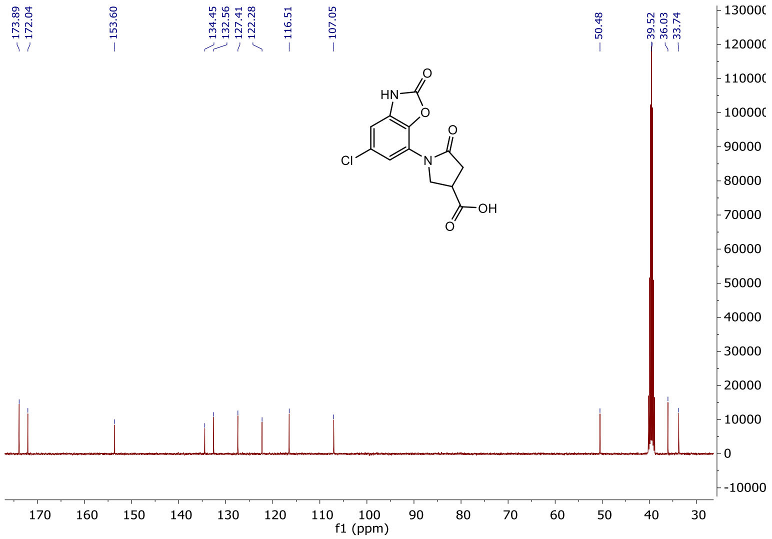

Figure S20. ^{13}C-NMR (400 MHz, DMSO-d_6) spectrum of **7**.

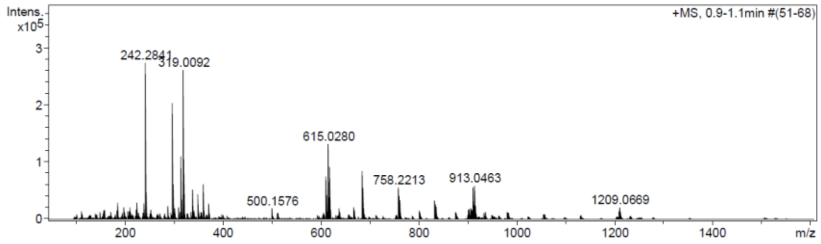

Meas. m/z	#	Formula	Score	m/z	err [ppm]	Mean err [ppm]	mSigma	rdb	e⁻ Conf	N-Rule
242.2841	1	C 16 H 36 N	100.00	242.2842	0.4	0.6	4.3	-0.5	even	ok
297.0273	1	C 12 H 10 Cl N 2 O 5	100.00	297.0273	0.0	0.2	17.2	8.5	even	ok

Figure S21. HRMS spectrum of **7**.

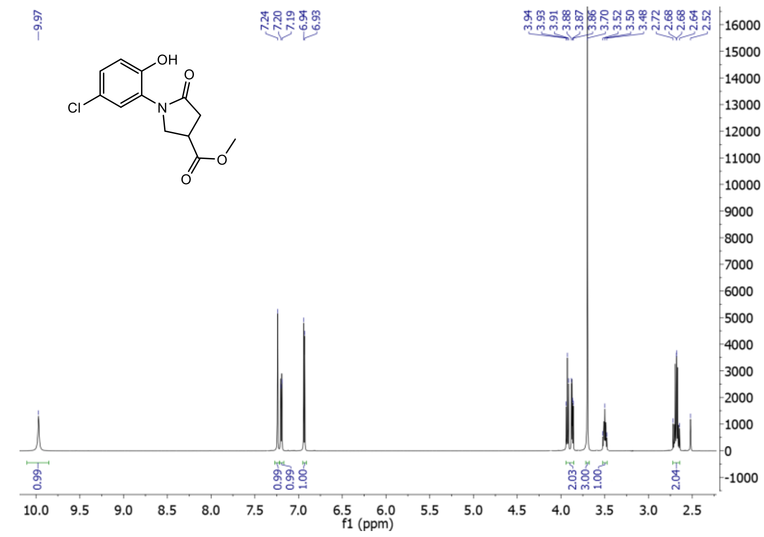

Figure S22. ^{1}H-NMR (400 MHz, DMSO-d_6) spectrum of **8**.

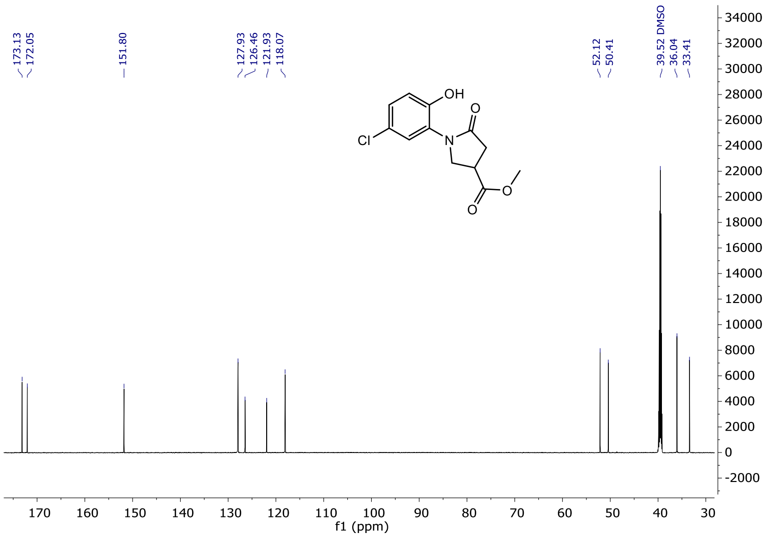

Figure S23. ^{13}C-NMR (400 MHz, DMSO-d_6) spectrum of **8**.

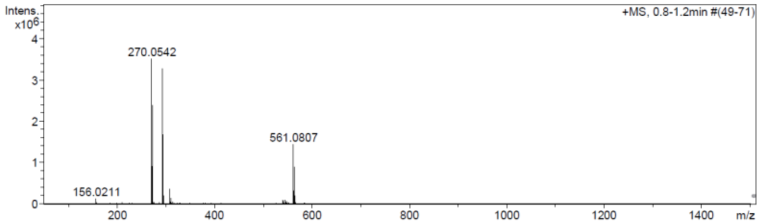

Figure S24. HRMS spectrum of **8**.

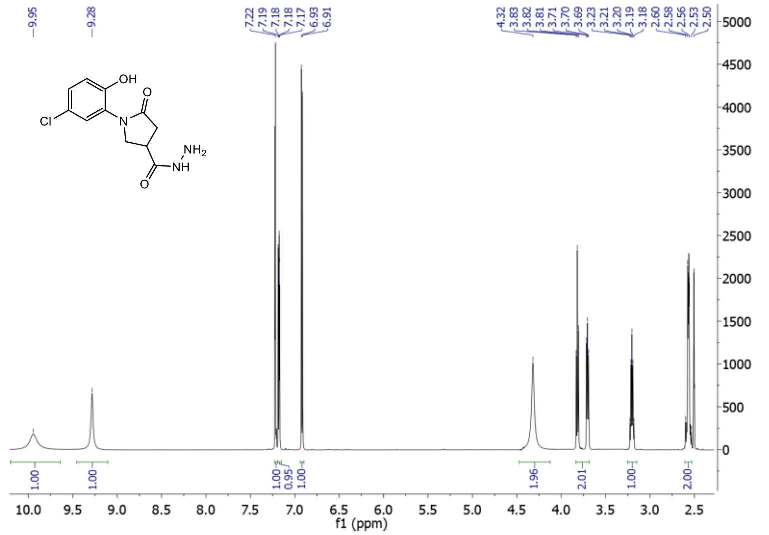

Figure S25. ^{1}H-NMR (400 MHz, DMSO-d_6) spectrum of **9**.

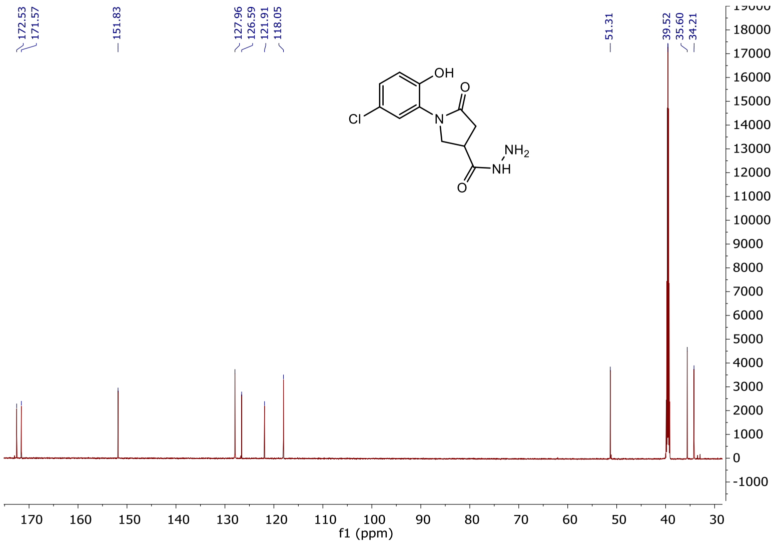

Figure S26. ^{13}C-NMR (400 MHz, DMSO-d_6) spectrum of **9**.

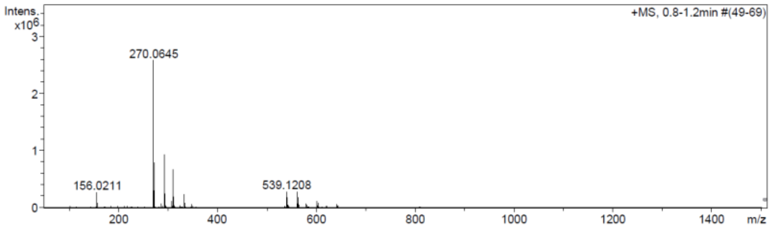

Meas. m/z	#	Formula	Score	m/z	err [ppm]	Mean err [ppm]	mSigma	rdb	e⁻ Conf	N-Rule
270.0645	1	C 11 H 13 Cl N 3 O 3	100.00	270.0640	-1.8	-1.4	17.3	6.5	even	ok
	2	C 17 H 8 N 3 O	23.22	270.0662	6.3	6.6	47.3	15.5	even	ok

Figure S27. HRMS spectrum of **9**.

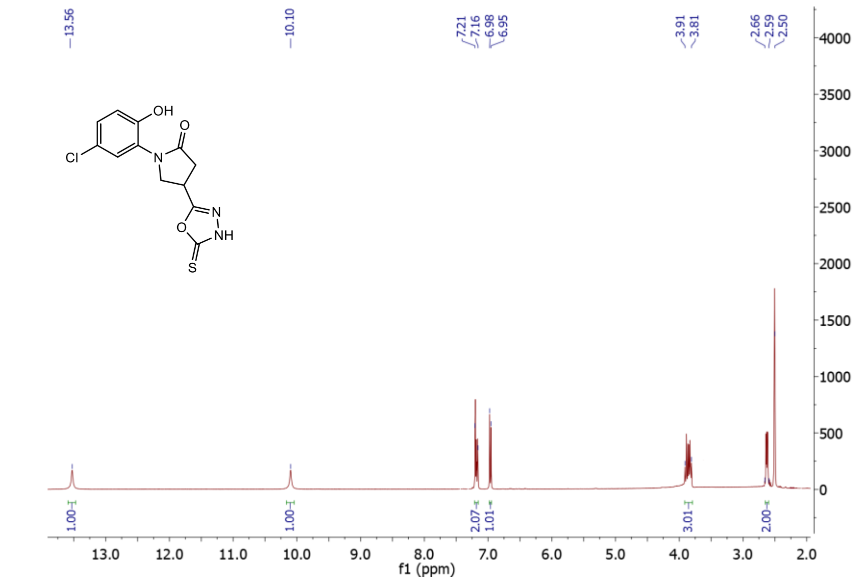

Figure S28. ^{1}H-NMR (400 MHz, DMSO-d$_6$) spectrum of **10**.

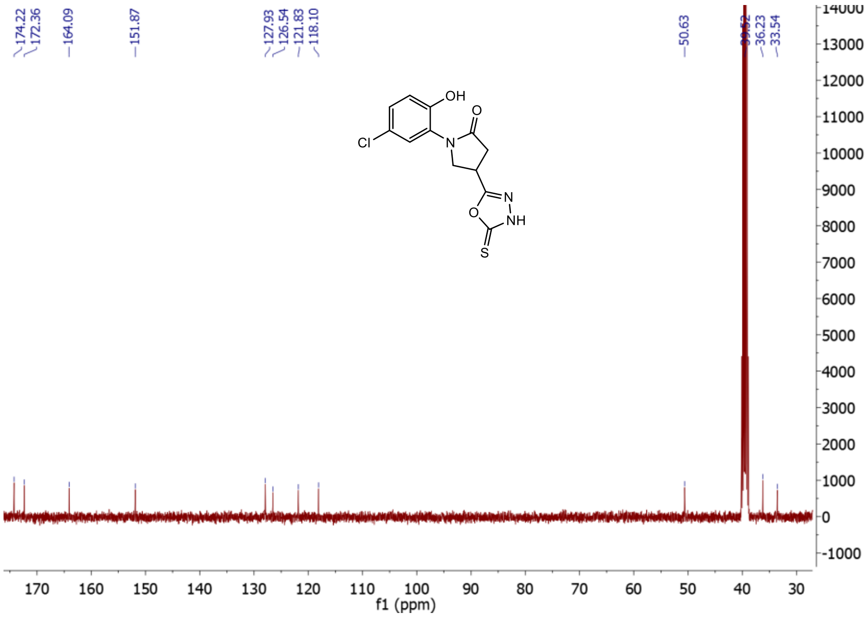

Figure S29. ^{13}C-NMR (400 MHz, DMSO-d$_6$) spectrum of **10**.

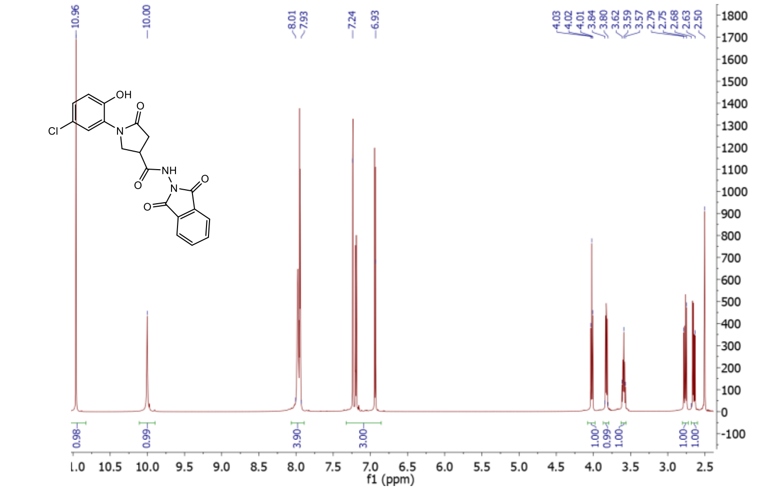

Figure S30. ^{1}H-NMR (400 MHz, DMSO-d$_6$) spectrum of **11**.

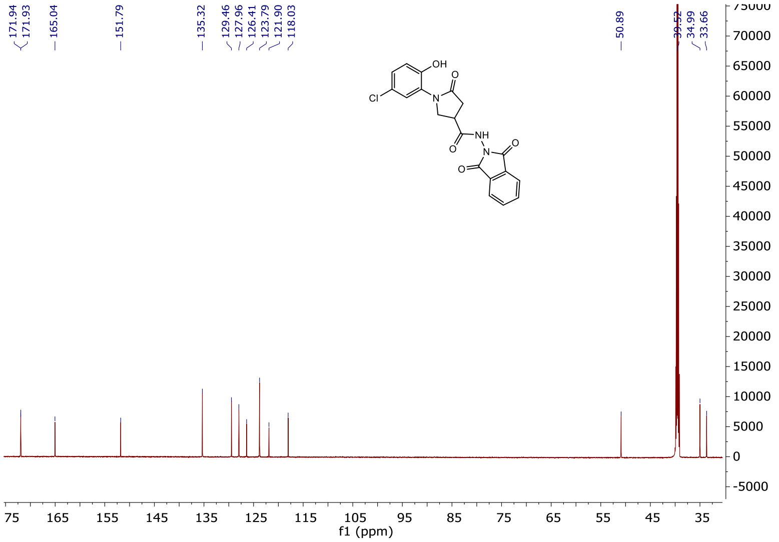

Figure S31. ^{13}C-NMR (400 MHz, DMSO-d$_6$) spectrum of **11**.

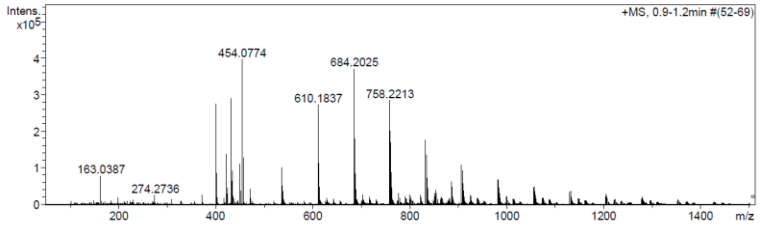

Meas. m/z	#	Formula	Score	m/z	err [ppm]	Mean err [ppm]	mSigma	rdb	e⁻ Conf	N-Rule
400.0693	1	C 19 H 15 Cl N 3 O 5	100.00	400.0695	0.5	0.7	18.0	13.5	even	ok
	2	C 20 H 11 Cl N 7 O	44.09	400.0708	3.8	4.0	22.1	18.5	even	ok
	3	C 20 H 10 N 5 O 5	37.49	400.0676	-4.1	-3.9	26.0	18.5	even	ok
	4	C 21 H 6 N 9 O	63.72	400.0690	-0.8	-0.6	35.3	23.5	even	ok

Figure S32. HRMS spectrum of **11**.

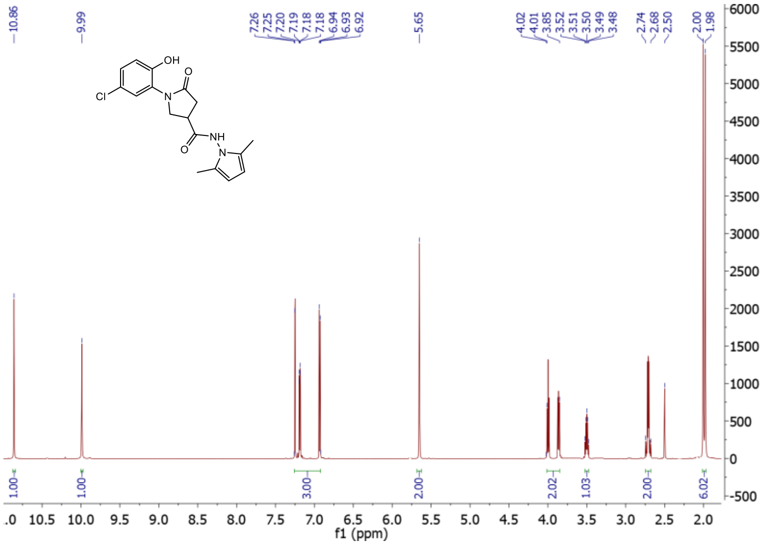

Figure S33. ^{1}H-NMR (400 MHz, DMSO-d_6) spectrum of **12**.

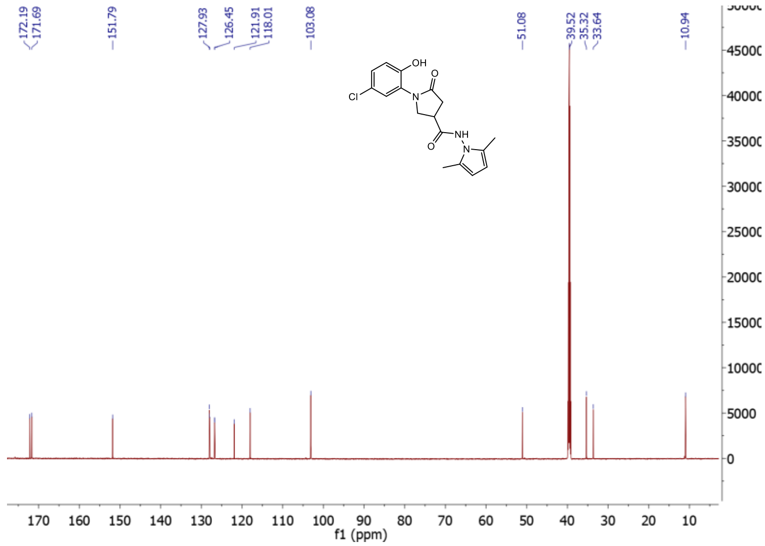

Figure S34. ^{13}C-NMR (400 MHz, DMSO-d_6) spectrum of **12**.

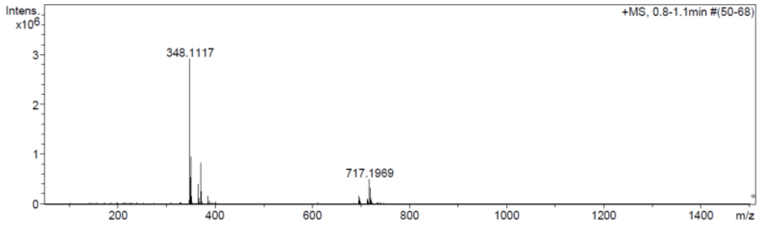

Meas. m/z	#	Formula	Score	m/z	err [ppm]	Mean err [ppm]	mSigma	rdb	e⁻ Conf	N-Rule
348.1117	1	C 17 H 19 Cl N 3 O 3	100.00	348.1109	-2.1	-1.6	11.2	9.5	even	ok
	2	C 23 H 14 N 3 O	26.86	348.1131	4.2	4.5	47.8	18.5	even	ok

Figure S35. HRMS spectrum of **12**.

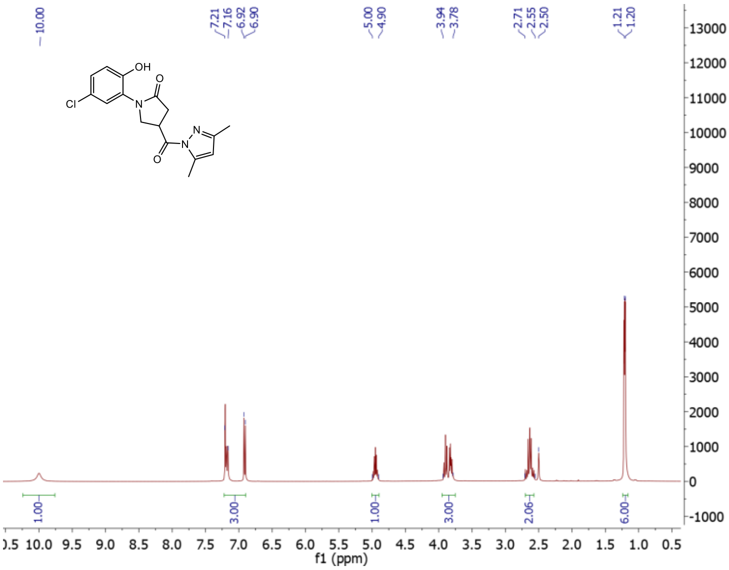

Figure S36. ^{1}H-NMR (400 MHz, DMSO-d_6) spectrum of **13**.

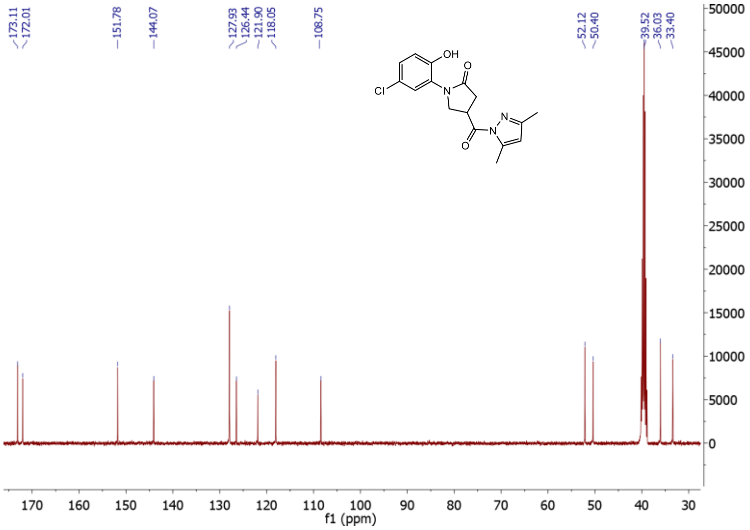

Figure S37. ^{13}C-NMR (400 MHz, DMSO-d_6) spectrum of **13**.

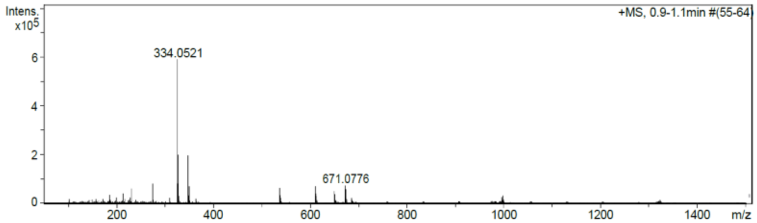

Meas. m/z	#	Formula	Score	m/z	err [ppm]	Mean err [ppm]	mSigma	rdb	e⁻ Conf	N-Rule
334.0521	1	C 10 H 9 N 6 O 7	90.25	334.0517	2.5	2.8	13.2	9.5	even	ok
	2	C 12 H 20 Cl O 6 N 2	60.47	334.0506	-3.7	-3.2	22.1	3.5	even	ok
	3	C 8 H 18 Cl O 11	55.88	334.0539	4.0	4.8	22.5	-0.5	even	ok
	4	C 9 H 13 N 2 O 11	85.96	334.0514	-1.6	-1.3	22.7	4.5	even	ok
	5	C 15 H 14 Cl N 3 O 3	100.00	334.0534	0.4	0.9	23.7	8.5	even	ok

Figure S38. HRMS spectrum of **13**.

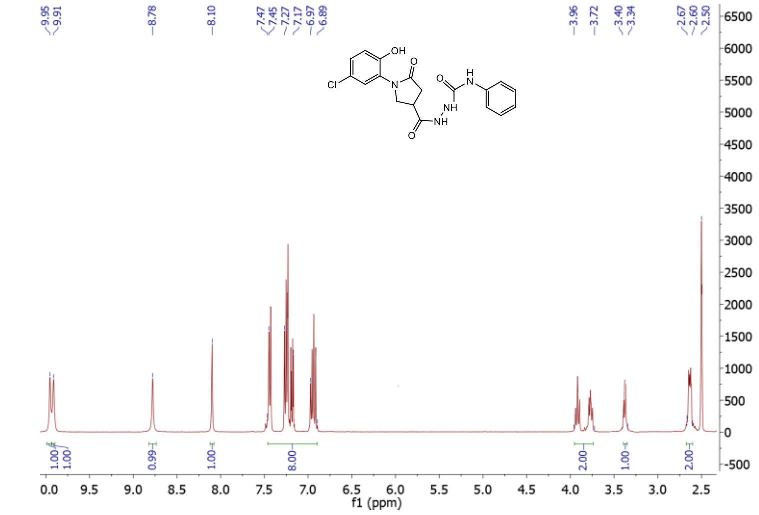

Figure S39. ^{1}H-NMR (400 MHz, DMSO-d_6) spectrum of **14**.

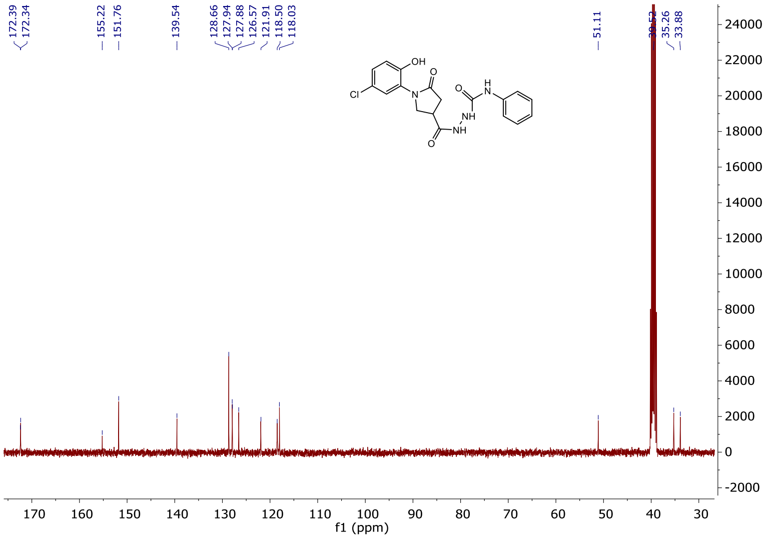

Figure S40. ^{13}C-NMR (400 MHz, DMSO-d_6) spectrum of **14**.

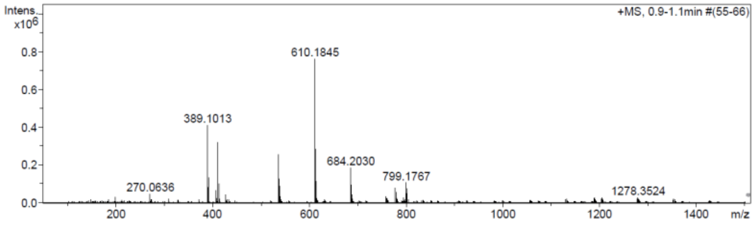

Meas. m/z	#	Formula	Score	m/z	err [ppm]	Mean err [ppm]	mSigma	rdb	e⁻ Conf	N-Rule
389.1013	1	C 18 H 18 Cl N 4 O 4	100.00	389.1011	-0.4	-0.2	11.9	11.5	even	ok

Figure S41. HRMS spectrum of **14**.

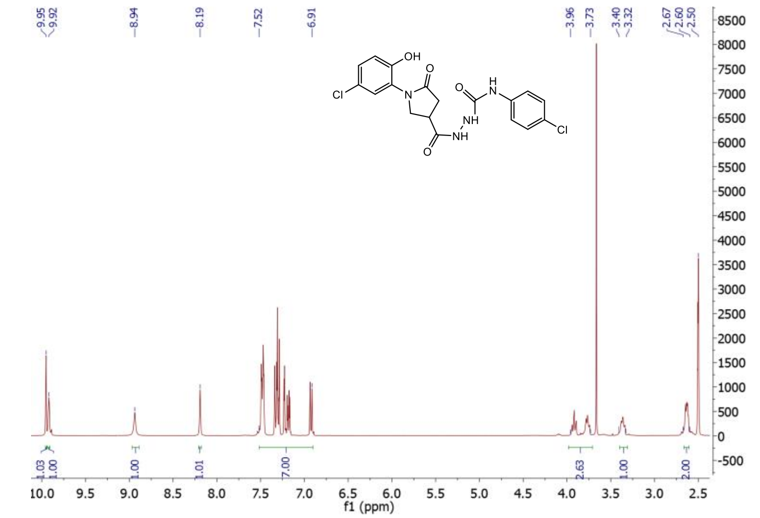

Figure S42. ^{1}H-NMR (400 MHz, DMSO-d$_6$) spectrum of **15**.

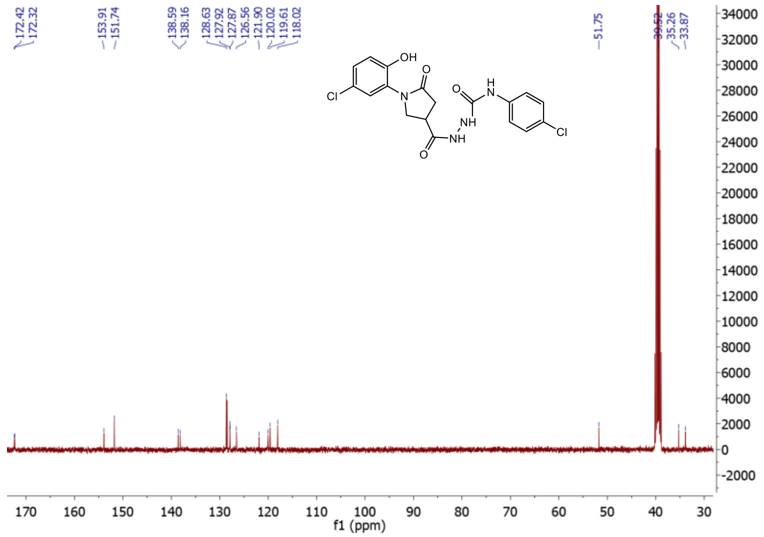

Figure S43. ^{13}C-NMR (400 MHz, DMSO-d$_6$) spectrum of **15**.

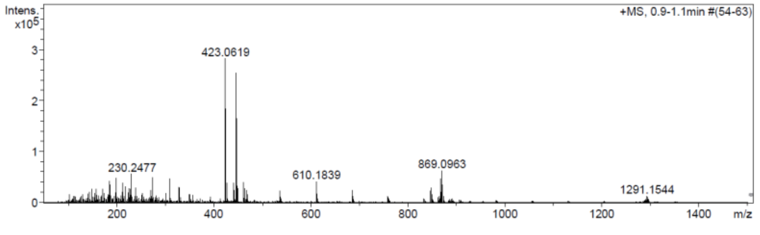

Meas. m/z	#	Formula	Score	m/z	err [ppm]	Mean err [ppm]	mSigma	rdb	e⁻ Conf	N-Rule
423.0619	1	C 18 H 17 Cl 2 N 4 O 4	100.00	423.0621	0.5	0.6	8.4	11.5	even	ok

Figure S44. HRMS spectrum of **15**.

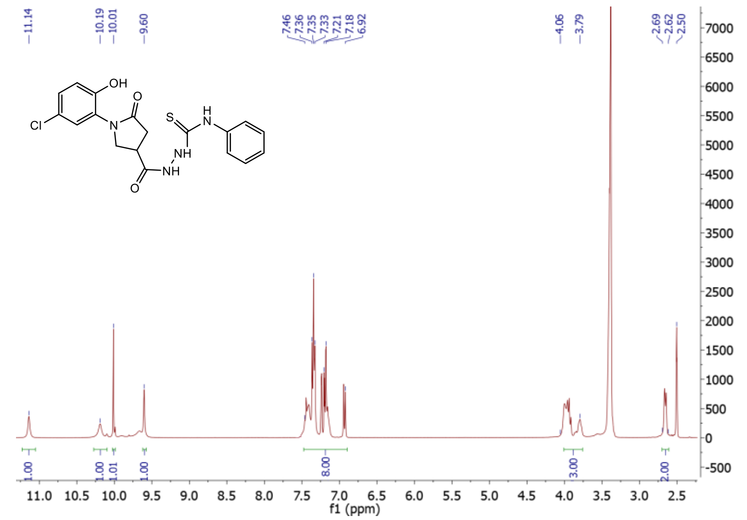

Figure S45. ^{1}H-NMR (400 MHz, DMSO-d6) spectrum of **16**.

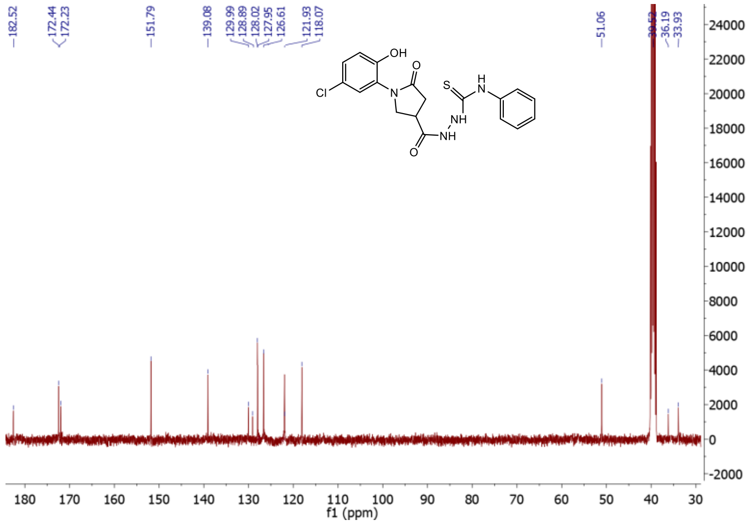

Figure S46. ^{13}C-NMR (400 MHz, DMSO-d6) spectrum of **16**.

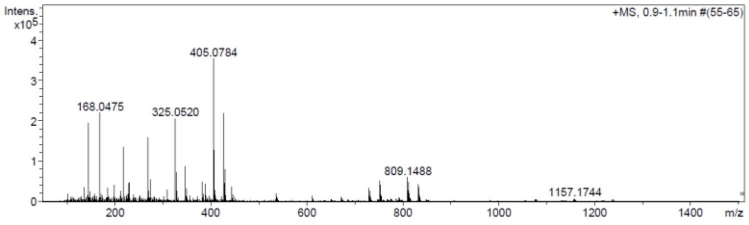

Meas. m/z	#	Formula	Score	m/z	err [ppm]	Mean err [ppm]	mSigma	rdb	e⁻ Conf	N-Rule
405.0784	1	C 16 H 9 N 10 O 4	40.04	405.0803	4.6	4.8	16.8	17.5	even	ok
	2	C 18 H 18 Cl N 4 O 3 S	100.00	405.0783	-0.4	-0.0	20.2	11.5	even	ok

Figure S47. HRMS spectrum of **16**.

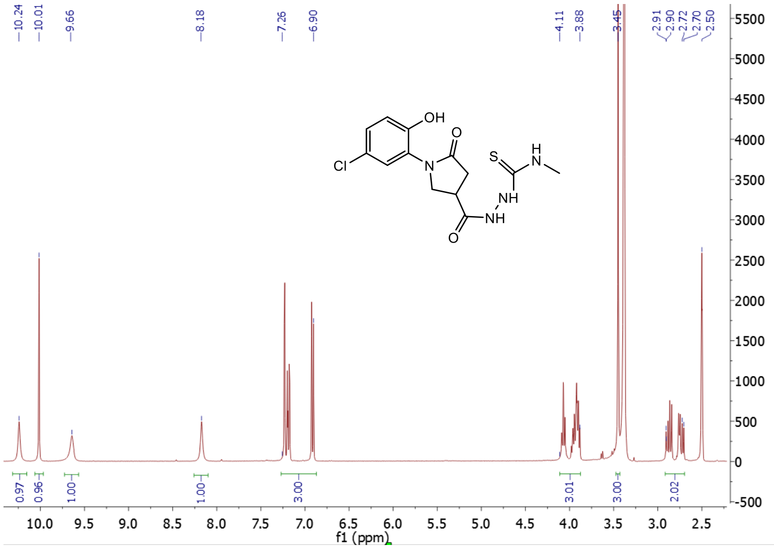

Figure S48. ^{1}H-NMR (400 MHz, DMSO-d$_6$) spectrum of **17**.

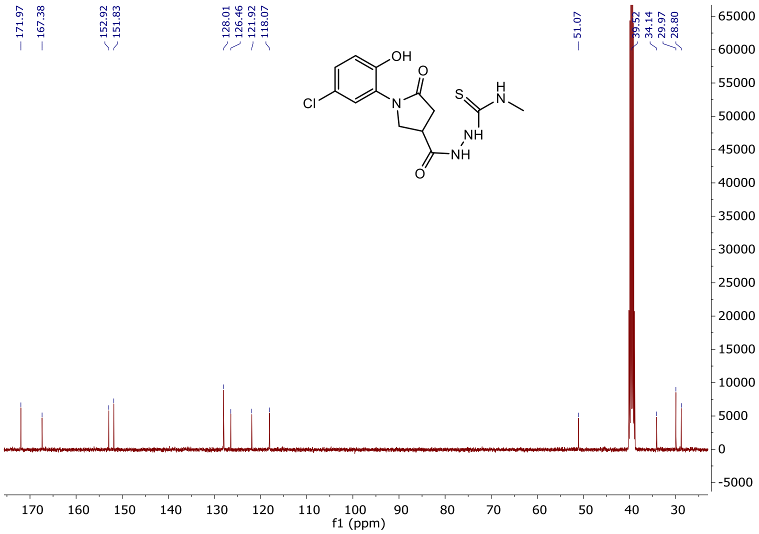

Figure S49. ^{13}C-NMR (400 MHz, DMSO-d$_6$) spectrum of **17**.

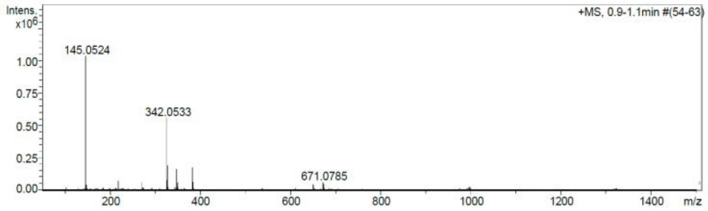

Meas. m/z	#	Formula	Score	m/z	err [ppm]	Mean err [ppm]	mSigma	rdb	e⁻ Conf	N-Rule
145.0524	1	C 4 H 9 N 4 S	100.00	145.0542	-0.8	-0.4	16.1	2.5	even	ok
	2	C 6 H 10 Cl N 2	0.24	145.0527	-11.5	-10.7	142.9	2.5	even	ok
342.0523	1	C 10 H 9 N 6 O 7	100.00	342.0607	1.3	1.6	13.1	9.5	even	ok
	2	C 11 H 5 N 10 O 3	43.61	342.0541	5.4	5.7	15.3	14.5	even	ok
	3	C 8 H 18 Cl O 11	64.86	342.0532	2.8	3.6	21.9	-0.5	even	ok
	4	C 9 H 13 N 2 O 11	64.03	342.0514	-2.8	-2.5	22.3	4.5	even	ok
	5	C 12 H 18 Cl O 6 S	42.03	342.0507	-4.9	-4.4	22.6	3.5	even	ok
	6	C 13 H 14 Cl N 4 O 2 S	84.92	342.0521	-0.8	-0.3	24.5	8.5	even	ok
	7	C 21 H 10 Cl N 2	45.20	342.0527	1.2	1.8	46.2	17.5	even	ok
347.0340	1	C 11 H 8 Cl N 10 S	100.00	347.0337	-0.8	-0.5	11.4	12.5	even	ok
	2	C 10 H 12 Cl N 6 O 4 S	45.05	347.0324	-4.7	-4.4	13.5	7.5	even	ok

Figure S50. HRMS spectrum of **17**.

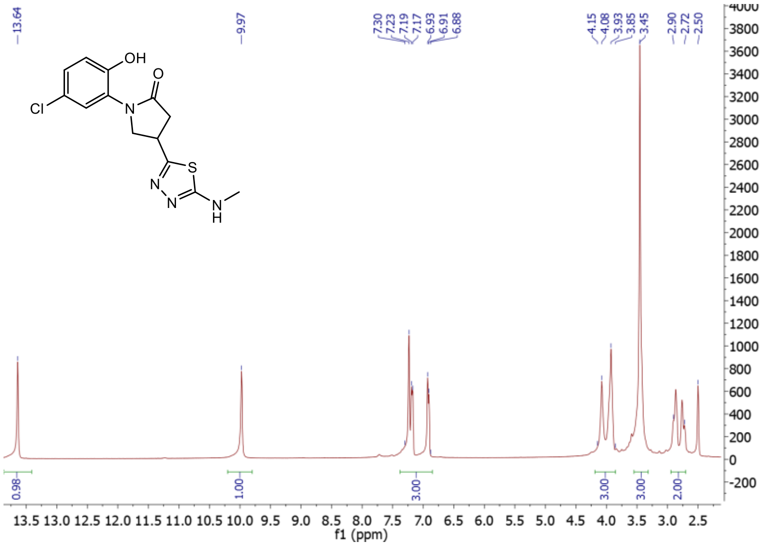

Figure S51. ^{1}H-NMR (400 MHz, DMSO-d$_6$) spectrum of **18**.

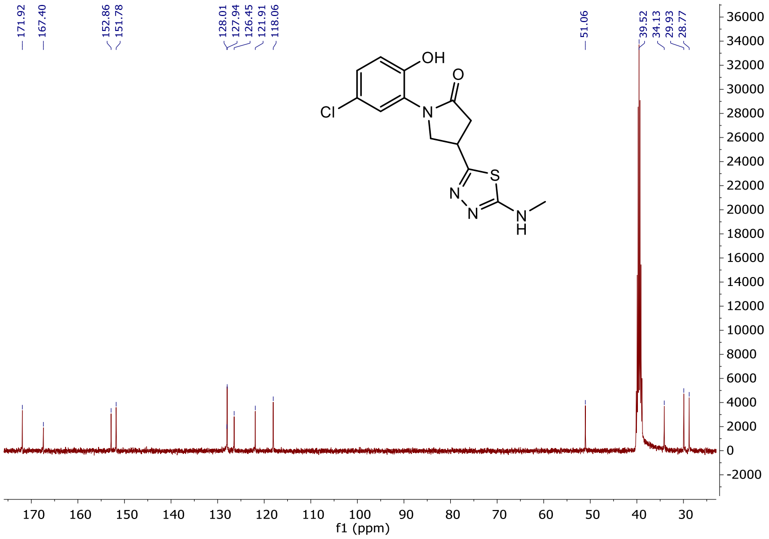

Figure S52. ^{13}C-NMR (400 MHz, DMSO-d$_6$) spectrum of **18**.

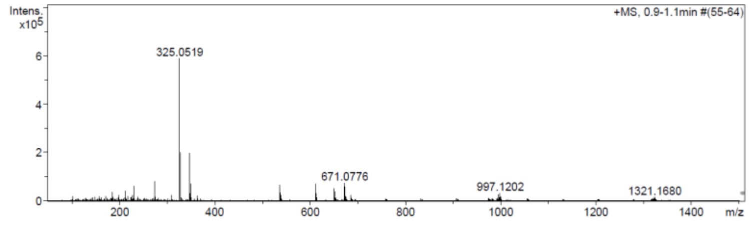

Meas. m/z	#	Formula	Score	m/z	err [ppm]	Mean err [ppm]	mSigma	rdb	e⁻ Conf	N-Rule
325.0519	1	C 10 H 9 N 6 O 7	90.25	325.0527	2.5	2.8	13.2	9.5	even	ok
	2	C 12 H 18 Cl O 6 S	60.47	325.0507	-3.7	-3.2	22.1	3.5	even	ok
	3	C 8 H 18 Cl O 11	55.88	325.0532	4.0	4.8	22.5	-0.5	even	ok
	4	C 9 H 13 N 2 O 11	85.96	325.0514	-1.6	-1.3	22.7	4.5	even	ok
	5	C 13 H 14 Cl N 4 O 2 S	100.00	325.0521	0.4	0.9	23.7	8.5	even	ok

Figure S53. HRMS spectrum of **18**.

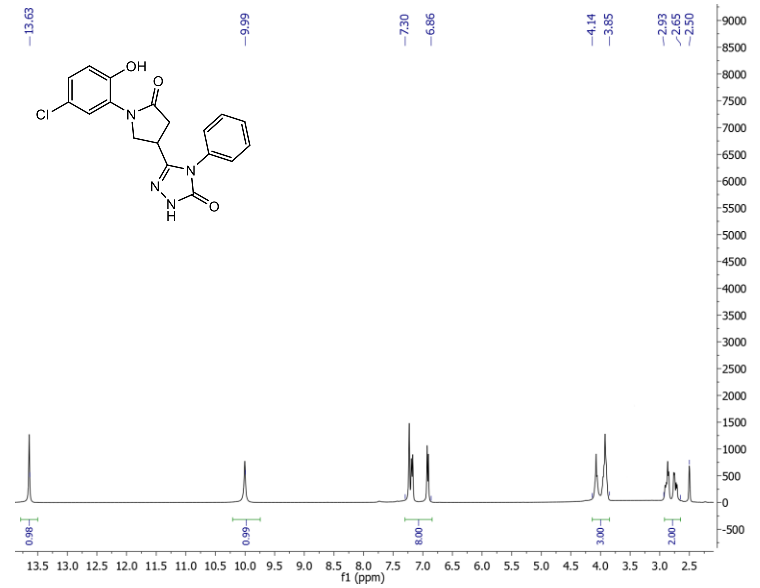

Figure S54. ^{1}H-NMR (400 MHz, DMSO-d6) spectrum of **19**.

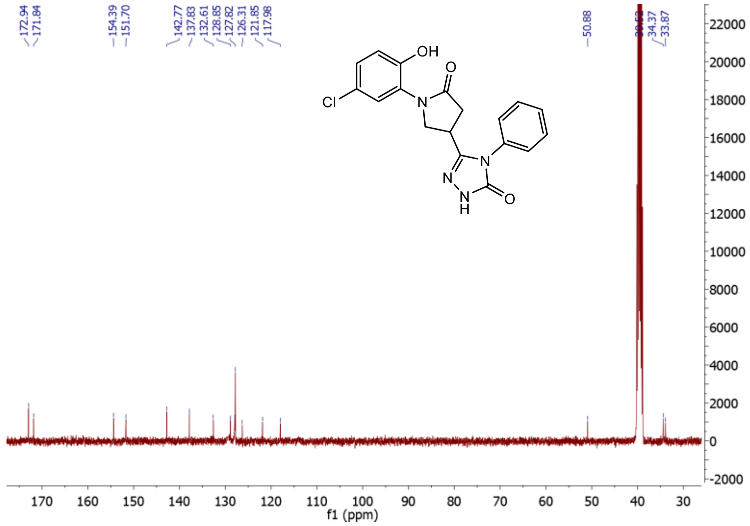

Figure S55. ^{13}C-NMR (400 MHz, DMSO-d6) spectrum of **19**.

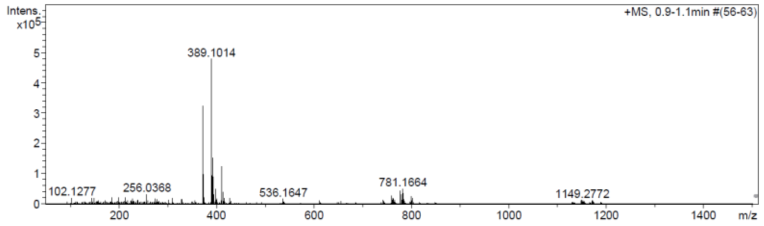

Meas. m/z	#	Formula	Score	m/z	err [ppm]	Mean err [ppm]	mSigma	rdb	e⁻ Conf	N-Rule
371.0907	1	C 17 H 20 Cl O 7	49.35	371.0892	-4.1	-4.2	20.7	7.5	even	ok
	2	C 18 H 16 Cl N 4 O 3	100.00	371.0905	-0.5	-0.6	21.8	12.5	even	ok

Figure S56. HRMS spectrum of **19**.

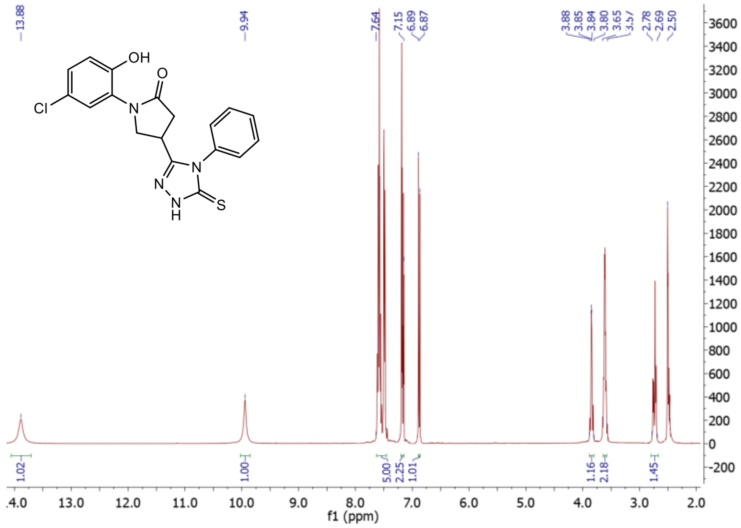

Figure S57. ^{1}H-NMR (400 MHz, DMSO-d6) spectrum of **20**.

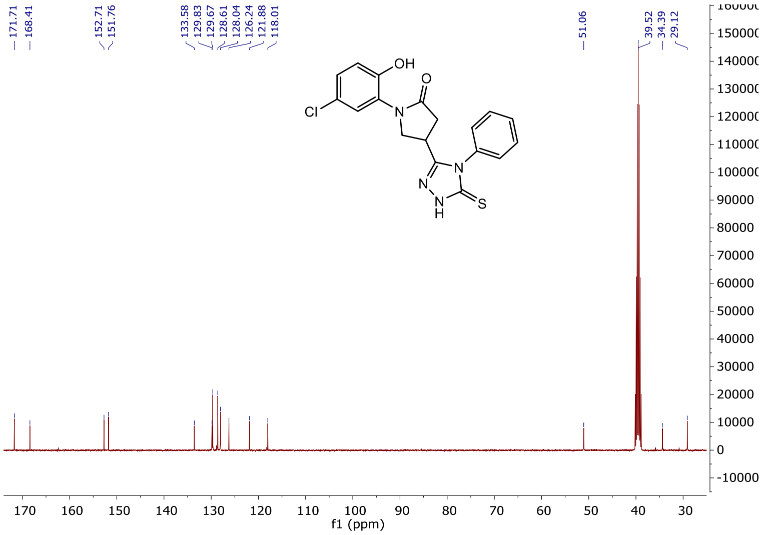

Figure S58. ^{13}C-NMR (400 MHz, DMSO-d6) spectrum of **20**.

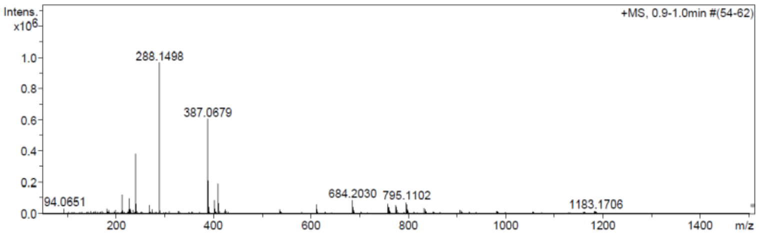

Meas. m/z	#	Formula	Score	m/z	err [ppm]	Mean err [ppm]	mSigma	rdb	e⁻ Conf	N-Rule
387.0679	1	C 16 H 7 N 10 O 3	43.36	387.0697	4.8	5.0	15.7	18.5	even	ok
	2	C 15 H 11 N 6 O 7	87.28	387.0684	1.3	1.6	21.0	13.5	even	ok
	3	C 18 H 16 Cl N 4 O 2 S	100.00	387.0677	-0.4	-0.1	22.2	12.5	even	ok

Figure S59. HRMS spectrum of **20**.

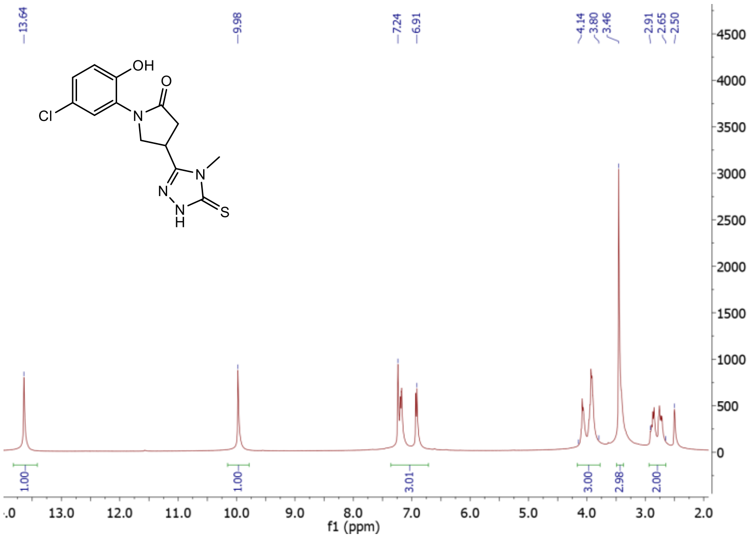

Figure S60. ^{1}H-NMR (400 MHz, DMSO-d_6) spectrum of **21**.

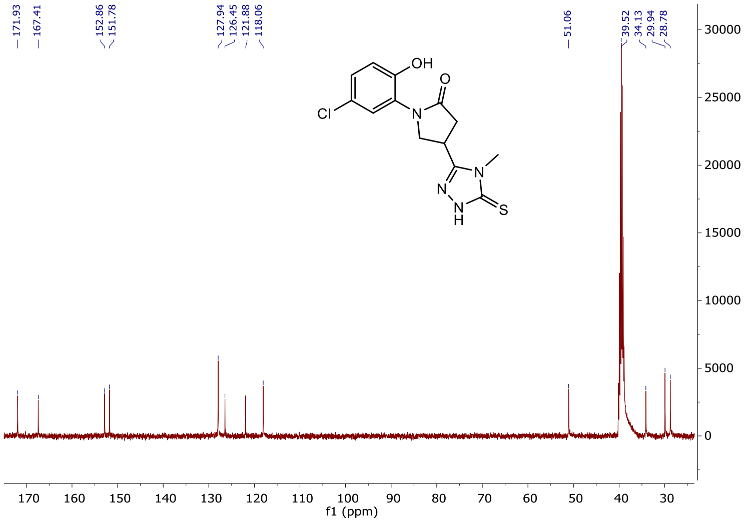

Figure S61. ^{13}C-NMR (400 MHz, DMSO-d_6) spectrum of **21**.

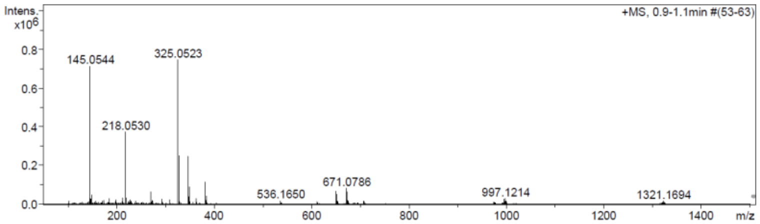

Meas. m/z	#	Formula	Score	m/z	err [ppm]	Mean err [ppm]	mSigma	rdb	e⁻ Conf	N-Rule
325.0523	1	C 10 H 9 N 6 O 7	100.00	325.0527	1.2	1.5	13.3	9.5	even	ok
	2	C 11 H 5 N 10 O 3	44.62	325.0541	5.3	5.6	14.6	14.5	even	ok
	3	C 12 H 18 Cl O 6 S	41.99	325.0507	-5.0	-4.5	21.8	3.5	even	ok
	4	C 9 H 13 N 2 O 11	62.21	325.0514	-2.9	-2.6	23.0	4.5	even	ok
	5	C 8 H 18 Cl O 11	63.70	325.0532	2.7	3.5	23.0	-0.5	even	ok
	6	C 13 H 14 Cl N 4 O 2 S	86.11	325.0521	-0.8	-0.4	23.3	8.5	even	ok

Figure S62. HRMS spectrum of **21**.

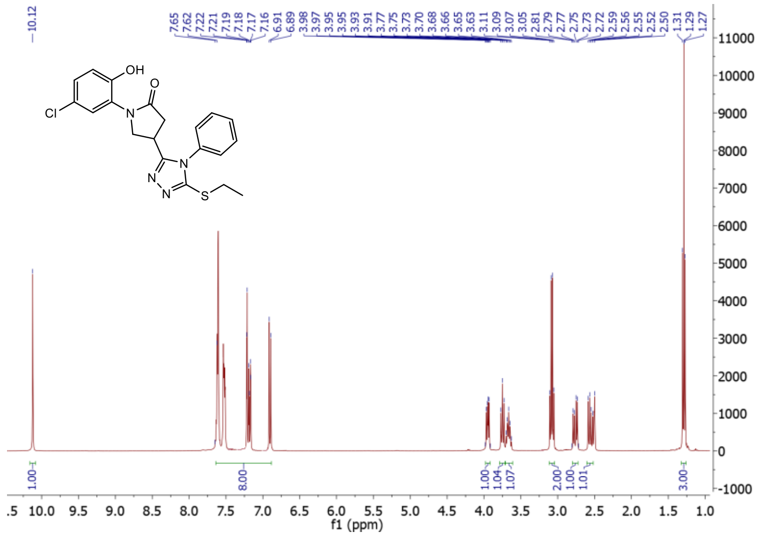

Figure S63. ¹H-NMR (400 MHz, DMSO-d₆) spectrum of **22**.

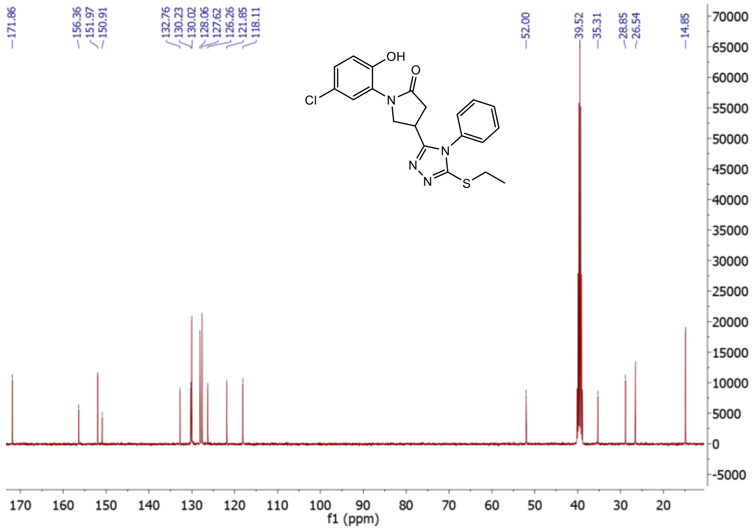

Figure S64. ¹³C-NMR (400 MHz, DMSO-d₆) spectrum of **22**.

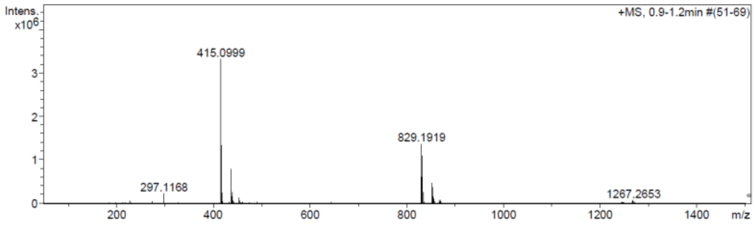

Meas. m/z	#	Formula	Score	m/z	err [ppm]	Mean err [ppm]	mSigma	rdb	e⁻ Conf	N-Rule
415.0999	1	C 20 H 20 Cl N 4 O 2 S	100.00	415.0990	-2.2	-1.6	8.1	12.5	even	ok

Figure S65. HRMS spectrum of **22**.

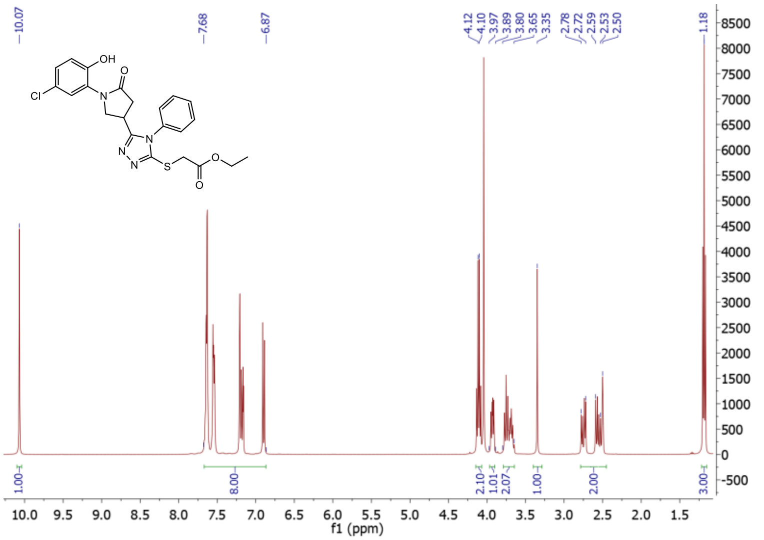

Figure S66. ^{1}H-NMR (400 MHz, DMSO-d_6) spectrum of **23**.

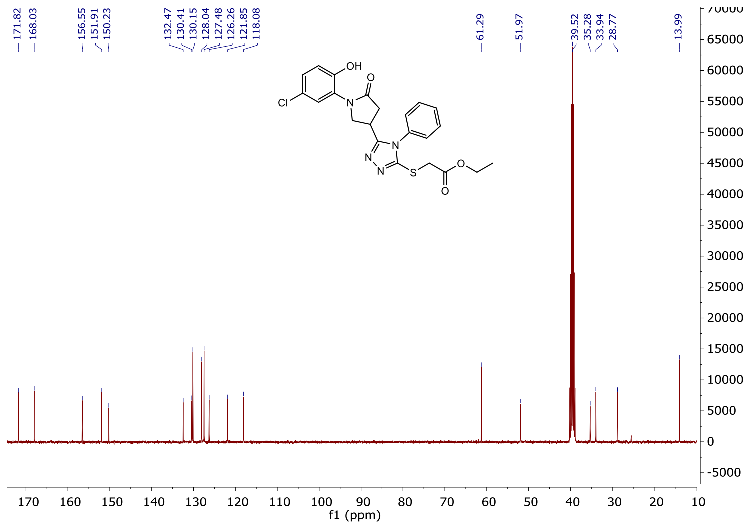

Figure S67. ^{13}C-NMR (400 MHz, DMSO-d_6) spectrum of **23**.

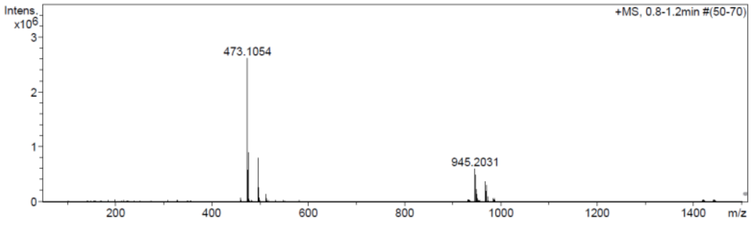

Meas. m/z	#	Formula	Score	m/z	err [ppm]	Mean err [ppm]	mSigma	rdb	e⁻ Conf	N-Rule
473.1054	1	C 15 H 14 Cl N 14 O 3	100.00	473.1056	0.6	1.0	5.9	15.5	even	ok
	2	C 18 H 22 Cl N 4 O 9	43.37	473.1070	3.4	3.9	10.2	9.5	even	ok
	3	C 14 H 18 Cl N 10 O 7	55.02	473.1043	-2.3	-1.8	16.0	10.5	even	ok
	4	C 17 H 26 Cl O 13	78.95	473.1056	0.6	1.1	18.0	4.5	even	ok
	5	C 16 H 9 N 16 O 3	37.99	473.1038	-3.3	-3.1	19.0	20.5	even	ok
	6	C 19 H 17 N 6 O 9	74.81	473.1052	-0.4	-0.2	22.0	14.5	even	ok
	7	C 18 H 21 N 2 O 13	33.89	473.1038	-3.3	-2.9	24.5	9.5	even	ok
	8	C 20 H 13 N 10 O 5	38.56	473.1065	2.4	2.6	30.4	19.5	even	ok
	9	C 22 H 22 Cl N 4 O 4 S	37.71	473.1045	-1.9	-1.6	36.7	13.5	even	ok

Figure S68. HRMS spectrum of **23**.

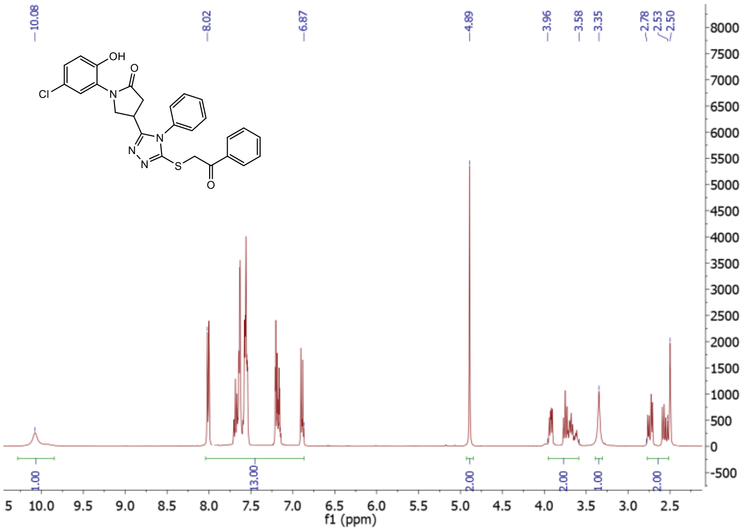

Figure S69. ^{1}H-NMR (400 MHz, DMSO-d$_6$) spectrum of **24**.

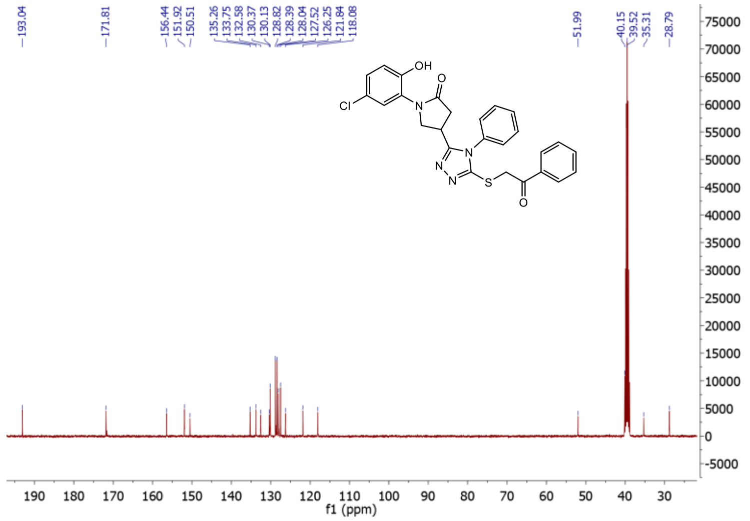

Figure S70. ^{13}C-NMR (400 MHz, DMSO-d$_6$) spectrum of **24**.

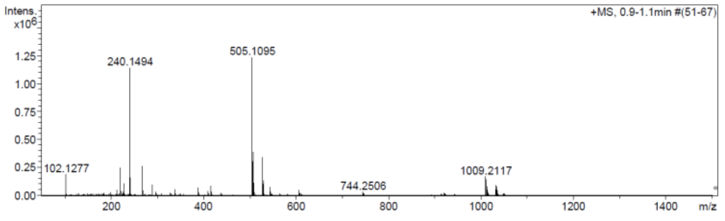

Meas. m/z	#	Formula	Score	m/z	err [ppm]	Mean err [ppm]	mSigma	rdb	e⁻ Conf	N-Rule
505.1095	1	C 19 H 14 Cl N 14 O 2	59.18	505.1107	2.4	2.6	19.9	19.5	even	ok
	2	C 18 H 18 Cl N 10 O 6	100.00	505.1094	-0.2	0.0	21.7	14.5	even	ok
	3	C 19 H 13 N 12 O 6	32.90	505.1076	-3.9	-3.7	24.0	19.5	even	ok
	4	C 21 H 26 Cl O 12	50.50	505.1107	2.4	2.7	26.7	8.5	even	ok
	5	C 22 H 21 N 2 O 12	70.24	505.1089	-1.2	-1.0	27.5	13.5	even	ok
	6	C 17 H 22 Cl N 6 O 10	42.34	505.1080	-2.9	-2.7	28.1	9.5	even	ok
	7	C 20 H 9 N 16 O 2	68.63	505.1089	-1.2	-1.1	28.3	24.5	even	ok
	8	C 23 H 17 N 6 O 8	63.56	505.1102	1.5	1.6	28.8	18.5	even	ok
	9	C 25 H 26 Cl O 7 S	30.33	505.1082	-2.5	-2.5	45.2	12.5	even	ok
	10	C 26 H 22 Cl N 4 O 3 S	49.56	505.1096	0.1	0.1	49.5	17.5	even	ok

Figure S71. HRMS spectrum of **24**.

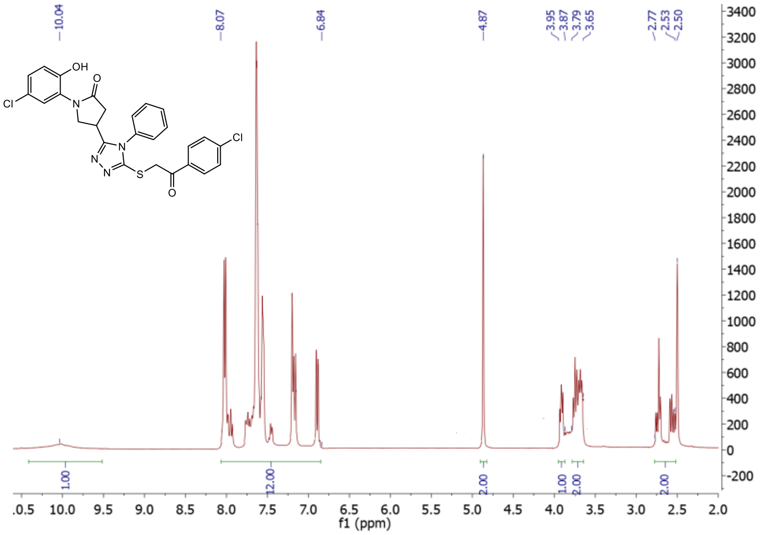

Figure S72. ^{1}H-NMR (400 MHz, DMSO-d6) spectrum of **25**.

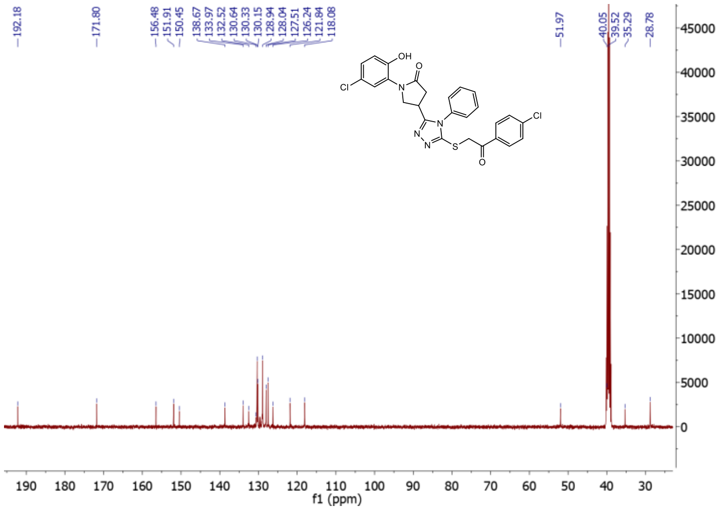

Figure S73. ^{13}C-NMR (400 MHz, DMSO-d6) spectrum of **25**.

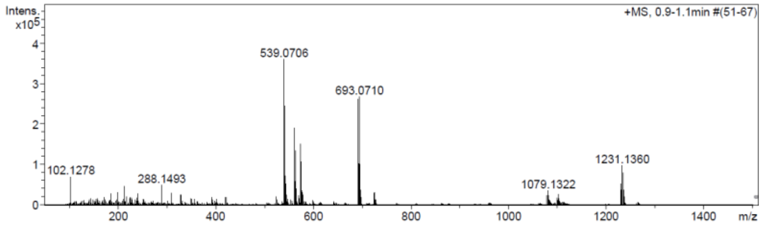

Meas. m/z	#	Formula	Score	m/z	err [ppm]	Mean err [ppm]	mSigma	rdb	e⁻ Conf	N-Rule
539.0706	1	C 19 H 13 Cl 2 N 14 O 2	61.60	539.0717	2.1	2.2	20.7	19.5	even	ok
	2	C 25 H 25 Cl 2 O 7 S	50.91	539.0693	-2.5	-2.3	24.3	12.5	even	ok
	3	C 26 H 21 Cl 2 N 4 O 3 S	100.00	539.0706	0.0	0.1	24.7	17.5	even	ok

Figure S74. HRMS spectrum of **25**.

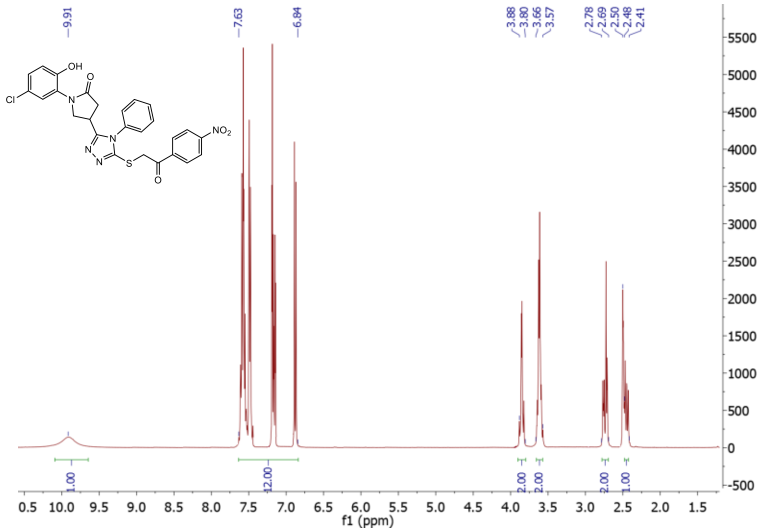

Figure S75. ^{1}H-NMR (400 MHz, DMSO-d6) spectrum of **26**.

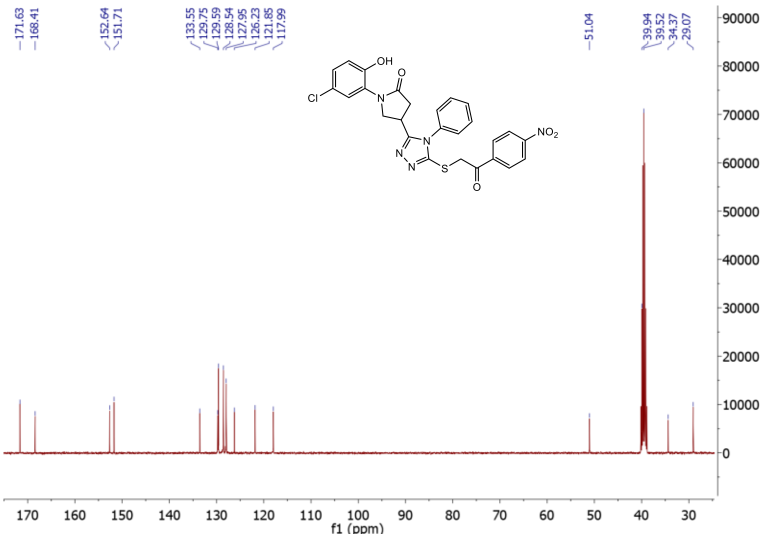

Figure S76. ^{13}C-NMR (400 MHz, DMSO-d6) spectrum of **26**.

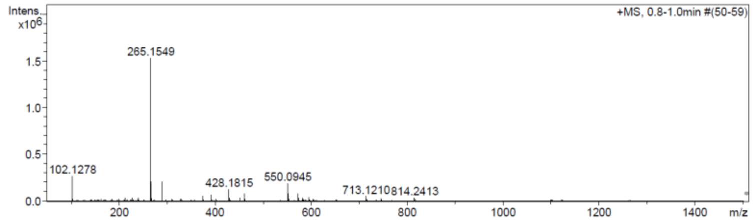

Meas. m/z	#	Formula	Score	m/z	err [ppm]	Mean err [ppm]	mSigma	rdb	e⁻ Conf	N-Rule
550.0945	1	C 26 H 21 Cl N 5 O 5 S	100.00	550.0946	0.3	0.3	11.7	18.5	even	ok

Figure S77. HRMS spectrum of **26**.
